# Epigenetic silencing of the liver‐specific lncRNA *LUNAR* promotes liver cancer progression via NOTCH activation

**DOI:** 10.1002/1878-0261.70301

**Published:** 2026-07-12

**Authors:** Se Ha Jang, Hyung Seok Kim, Geum Ok Baek, Moon Gyeong Yoon, Su In Lee, Jee‐Yeong Jeong, Ji Eun Han, Soon Sun Kim, Jae Youn Cheong, Jung Woo Eun

**Affiliations:** ^1^ Department of Gastroenterology Ajou University School of Medicine Suwon South Korea; ^2^ Department of Biomedical Sciences Ajou University Graduate School of Medicine Suwon South Korea; ^3^ Department of Biochemistry Kosin University College of Medicine Busan South Korea; ^4^ Section of Hematology, Department of Internal Medicine and Yale Cancer Center Yale University School of Medicine New Haven Connecticut USA

**Keywords:** epithelial–mesenchymal transition, hepatocellular carcinoma, long noncoding RNA, LUNAR, metastasis

## Abstract

While long noncoding RNAs (lncRNAs) play critical roles in hepatocellular carcinoma (HCC) pathogenesis, the regulatory mechanisms governing liver‐specific lncRNAs remain poorly defined. Here, we systematically analyzed staging‐specific transcriptomic datasets to identify functionally relevant lncRNAs involved in HCC progression. We identified a liver‐specific lncRNA, termed *LUNAR* (Liver‐specific Upregulated Non‐coding RNA as Down‐Regulator in HCC), reflecting its high expression in normal liver tissue and its functional role in restraining tumor progression in HCC. Analyses across multiple independent patient cohorts revealed that *LUNAR* expression correlated with clinical outcomes in HCC. Functional assays demonstrated that restoration of *LUNAR* expression suppressed cell migration, invasion, and *in vivo* metastasis, without affecting cell proliferation. Mechanistically, *LUNAR* overexpression was associated with attenuation of NOTCH signaling, while its downregulation in tumors was linked to epigenetic silencing via promoter hypermethylation. Collectively, these findings identify *LUNAR* as a liver‐enriched, epigenetically regulated lncRNA with metastasis‐restraining functions. This therefore supports the potential of *LUNAR* as a tissue‐associated biomarker and therapeutic target in HCC.

## Graphical abstract

LUNAR is a liver‐specific long noncoding RNA (lncRNA) that is highly expressed in normal liver but becomes epigenetically silenced in hepatocellular carcinoma through promoter hypermethylation. Loss of LUNAR is associated with NOTCH activation, epithelial–mesenchymal transition, and metastasis, whereas restoring LUNAR restrains metastatic progression.

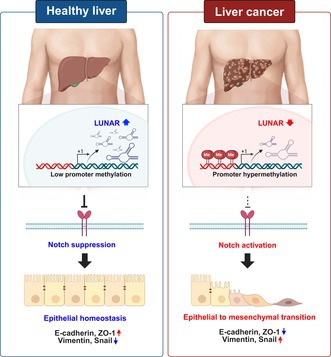

**Abbreviations**
5‐Aza: 5‐aza‐2′‐deoxycytidine
AASLD: American Association for the Study of Liver Diseases
AFP: alpha‐fetoprotein
aHCC: advanced hepatocellular carcinoma
ALT: alanine aminotransferase
ANOVA: analysis of variance
AST: aspartate aminotransferase
AUC: area under the curve
BCA: bicinchoninic acid
BCLC: Barcelona Clinic Liver Cancer
CH: chronic hepatitis
CI: confidence intervals
DSS: disease‐specific survival
EDTA: ethylenediaminetetraacetic acid
eHCC: early hepatocellular carcinoma
EMT: epithelial–mesenchymal transition
EV: extracellular vesicle
EX‐LUNAR: LUNAR overexpression vector
FPKM: Fragments Per Kilobase of transcript per Million mapped reads
GEO: Gene Expression Omnibus
GSEA: Gene set enrichment analysis
GTEx: Genotype‐Tissue Expression
H&E: hematoxylin and eosin
HBV: hepatitis B virus
HCC: hepatocellular carcinoma
HCV: hepatitis C virus
IHC: immunohistochemistry
LC: liver cirrhosis
lncRNAs: long noncoding RNAs
LUNAR: Liver‐specific Upregulated Non‐coding RNA as Down‐Regulator in HCC
MALAT1: metastasis‐associated lung adenocarcinoma transcript 1
MTT: 3‐(4,5‐dimethylthiazol‐2‐yl)‐2,5‐diphenyltetrazolium bromide
mUICC: modified Union for International Cancer Control
NES: normalized enrichment score
NICD: NOTCH intracellular domain
NL: normal liver
NT: non‐tumor
OS: overall survival
PBS: phosphate‐buffered saline
PIVKA‐II: Prothrombin‐induced by vitamin K absence or antagonist‐II
PVDF: polyvinylidene difluoride
qMSP: quantitative methylation‐specific polymerase chain reaction
qRT‐PCR: quantitative real‐time polymerase chain reaction
RIPA: radio immunoprecipitation assay
ROC: (receiver operating characteristic)
RT: room temperature
SD: standard deviation
SDS/PAGE: sodium dodecyl sulfate polyacrylamide gel electrophoresis
siNCs: small interfering negative‐control RNA duplexes
siRNAs: small interfering RNA duplexes
T: tumor
TCGA_LIHC: The Cancer Genome Atlas_Liver Hepatocellular Carcinoma
TET: ten–eleven translocation methylcytosine dioxygenase
UALCAN: University of Alabama at Birmingham Cancer Data Analysis Portal

## 1 Introduction

Long noncoding RNAs (lncRNAs) have emerged as critical regulators of gene expression in cancer, with tissue‐restricted expression patterns and dynamic regulation making them attractive candidates for both biomarker development and mechanism‐based therapies [1, 2]. Their ability to modulate transcriptional programs through chromatin remodeling, protein scaffolding, or microRNA sequestration has positioned lncRNAs as functionally diverse effectors of tumor biology [3, 4, 5, 6].

Despite these advances, the repertoire of liver‐enriched lncRNAs that are progressively silenced during the transition from chronic liver disease to hepatocellular carcinoma (HCC) remains incompletely defined [7]. In particular, lncRNAs that are progressively lost during malignant transformation represent an underexplored class of potential tumor suppressors and liver‐specific biomarkers. Systematic identification of such transcripts is therefore essential for illuminating novel tumor‐suppressive circuits and for developing liver‐specific biomarkers that may improve detection and characterization of HCC.

To this end, we identified *RP11‐252E2.2*, hereafter designated **L**iver‐specific **U**pregulated **N**on‐coding RN**A** as Down‐**R**egulator in HCC (*LUNAR*), as the most consistently and profoundly downregulated lncRNA across the spectrum from normal liver (NL) to chronic hepatitis (CH), liver cirrhosis (LC), early HCC (eHCC), and advanced HCC (aHCC). *LUNAR* was strikingly liver‐restricted in The Genotype‐Tissue Expression (GTEx) atlas, and its progressive loss was correlated with poor overall survival (OS) and disease‐specific survival (DSS). Receiver operating characteristic (ROC) analyses across multiple independent cohorts yielded area under the curve (AUC) values of 0.84–0.92 for distinguishing HCC from non‐tumor (NT) controls. These findings nominate *LUNAR* as a tissue‐associated biomarker candidate with potential diagnostic and prognostic relevance in HCC.

Furthermore, preliminary mechanistic data indicated that transient overexpression of *LUNAR* in HCC cells suppressed epithelial‐to‐mesenchymal transition (EMT) characteristics and angiogenic potential, findings that were associated with attenuation of NOTCH pathway activity—processes integral to metastasis and intrahepatic spread. Conversely, promoter hypermethylation and reduced ten–eleven translocation methylcytosine dioxygenase (TET)‐mediated demethylation may contribute to the silencing of *LUNAR* transcription in tumors, and treatment with the DNA‐methyltransferase inhibitor 5‐azacytidine (5‐Aza') restored its expression, suggesting an epigenetic mechanism of repression.

On the basis of these findings, we propose that *LUNAR* functions as a metastasis‐restraining regulator in HCC. Its progressive downregulation and liver‐enriched expression profile support its potential as a tissue‐associated biomarker candidate with diagnostic and prognostic relevance. These findings provide insight into the role of liver‐specific lncRNAs in HCC progression and suggest that *LUNAR* may represent a promising target for further mechanistic and translational investigation.

## 2 Materials and methods

### 2.1 Public dataset resources

To investigate the expression and clinical relevance of the long noncoding RNA *LUNAR* in HCC, transcriptomic data were obtained from The Cancer Genome Atlas_Liver Hepatocellular Carcinoma (TCGA_LIHC; https://cancergenome.nih.gov) and the Gene Expression Omnibus (GEO; https://www.ncbi.nlm.nih.gov/geo/) database. Additionally, *LUNAR*'s normal tissue‐specific expression patterns were assessed using RNA‐seq data from the GTEx Portal (https://gtexportal.org/home/), including datasets GSE114564, GSE77314, and GSE124535 [8]. Expression values of *LUNAR* were normalized and log2‐transformed [log_2_(Fragments Per Kilobase of transcript per Million mapped reads [FPKM] + 1)] for all analyses. The University of Alabama at Birmingham Cancer Data Analysis Portal (UALCAN) portal (https://ualcan.path.uab.edu/) was used to compare *LUNAR* expression across different tissue types and cancer stages to assess the prognostic value of *LUNAR* [9, 10]. Kaplan–Meier survival analysis was conducted using the TCGA_LIHC cohort, evaluating both OS and DSS. DSS was defined as the time from curative treatment to death specifically attributable to HCC.

### 2.2 Sample collection and clinical definitions

Tissue samples and associated clinical data were obtained from HCC patients who underwent surgical resection at Ajou University Hospital (Suwon, South Korea). The cohort consisted of 100 paired tumor (T) and adjacent non‐tumor (NT) liver tissues, which were snap‐frozen and stored in the institutional biobank. Detailed clinicopathological information for all patients is provided in Table 1.

**Table 1:** Clinicopathological characteristics of 100 patients in the tissue cohort. AFP, α‐fetoprotein; ALT, alanine aminotransferase; AST, aspartate aminotransferase; HBV, hepatitis B virus; HCV, hepatitis C virus; PIVKA‐II, prothrombin‐induced by vitamin K absence or antagonist‐II; SD, standard deviation; UICC, Union for International Cancer Control.

Variables	*n* = 100
Age (years), mean ± SD	56.4 ± 9.95
Male sex, *n* (%)	78 (78.0)
Etiology, *n* (%)	
HBV	93 (93.0)
HCV	5 (5.0)
Alcohol	1 (1.0)
HBV + Alcohol	1 (1.0)
Cirrhosis, *n* (%)	49 (49.0)
AST (IU·L^−1^), mean ± SD	50.0 ± 92.9
ALT (IU·L^−1^), mean ± SD	44.0 ± 55.9
Platelet (× 10^3^·μL^−1^), mean ± SD	177.4 ± 3.5
AFP (ng·mL^−1^), mean ± SD	2707.5 ± 8382.3
PIVKA‐II (mAU·mL^−1^), mean ± SD	7309.8 ± 17826.3
Albumin (g·dL^−1^), mean ± SD	4.4 ± 0.67
Total bilirubin (mg·dL^−1^), mean ± SD	0.76 ± 0.76
Creatinine (mg·dL^−1^), mean ± SD	0.94 ± 0.27
Sodium (mmol·L^−1^), mean ± SD	139.5 ± 1.4
Macrovascular invasion, *n* (%)	24 (32.4)
Lymph node metastasis, *n* (%)	1 (1.3)
Distant metastasis, *n* (%)	5 (6.6)
Modified UICC stage, *n* (%)	
I	27 (27.0)
II	37 (37.0)
III	23 (23.0)
IVA	6 (6.0)
IVB	7 (7.0)

HCC was diagnosed according to the American Association for the Study of Liver Diseases (AASLD) guidelines and staged using both the modified Union for International Cancer Control (mUICC) and the Barcelona Clinic Liver Cancer (BCLC) classification systems. Early‐stage HCC was defined as a solitary tumor less than 2 cm in diameter and corresponding to mUICC Stage I.

### 2.3 RNA isolation from paired HCC tissues and cell lines

Pairs of HCC tumors and adjacent non‐cancerous tissues were obtained from patients who underwent hepatectomy at Ajou University Hospital. Tissue samples were aliquoted into cryovials and stored at −80 °C until use. The cryopreserved tissue samples were homogenized using a mortar and pestle and coated with liquid nitrogen during grinding. Upon reaching a powdery texture, the samples were homogenized using QIAzol (Qiagen, Hilden, Germany). Total RNA was isolated from cell lines, according to the manufacturer's instructions, using QIAzol reagent.

### 2.4 Quantitative real‐time polymerase chain reaction (qRT‐PCR)

5× PrimeScript™ RT Master Mix (Takara Bio, Shiga, Japan) was used to synthesize cDNA from 500 ng of total RNA under the following conditions: 37 °C for 15 min, 85 °C for 5 s, and 4°C. AmfiSure qGreen Q‐PCR Master Mix (GenDEPOT, Barker, TX, USA) was used to conduct qRT‐PCR on a CFX Connect Real‐Time PCR Detection System (Bio‐Rad Laboratories, Hercules, CA, USA). HMBS was used as an endogenous reference.

The primer sequences were as follows: *LUNAR*: forward, 5′‐GATGCTGGCCAGTACAA‐3′ and reverse, 5′‐TGCATGACCACTGGCTA‐3′; *TET1*: forward, 5′‐CTCAAGACCTTGCCTCTTCTC‐3′ and reverse, 5′‐TGCTCACTGTCTGACCAATAC‐3′; *TET2*: forward, 5′‐CAAACTCTACTCGGAGCTTACC‐3′ and reverse, 5′‐CTTCCTTGGGATCTTGCTTCT‐3′; *HEY1*: forward, 5′‐GCTTTTGAGAAGCAGGGATCT‐3′ and reverse, 5′‐CCTTTCCCTCCTGCCGTATG‐3′; *HES1*: forward, 5′‐GGCTAAGGTGTTTGGAGGCT‐3′ and reverse, 5′‐GTGTAGACGGGGATGACAGG‐3′; *HES4*: forward, 5′‐AGCACCGCAAGTCCTCCAA‐3′ and reverse, 5′‐CTCTTTTCTGAGGGCGTCCA‐3′; *MALAT1*: forward, 5′‐GAATTGCGTCATTTAAAGCCTAGTT‐3′ and reverse, 5′‐GTTTCATCCTACCACTCCCAATTAAT‐3′; *GAPDH*: forward, 5′‐AGTATGACAACAGCCTCAAG‐3′ and reverse, 5′‐TCATGAGTCCTTCCACGATA‐3′; and *HMBS*: forward, 5′‐GCTCAGATAGCATACAAGAG‐3′ and reverse, 5′‐ACGAGCAGTGATGCCTACC‐3′. PCR was conducted under the following conditions: 95 °C for 2 min; 40 cycles at 95 °C for 15 s, 58–62 °C for 34 s, and 72 °C for 30 s; followed by a dissociation stage at 95 °C for 10 s, 65 °C for 5 s, and 95 °C for 5 s. The relative expression was determined using the relative standard curve method (2^−ΔΔCt^). All experiments were performed at least in triplicate.

### 2.5 Cell culture and transfection

The normal hepatocyte cell line MIHA (RRID:CVCL_SA11) was kindly provided by Dr. Roy‐Chowdhury (Albert Einstein College of Medicine, Bronx, NY, USA). The human HCC cell lines (Hep3B (RRID:CVCL_0326), Huh‐7 (RRID:CVCL_0336), PLC/PRF/5 (RRID:CVCL_0485), SNU368 (RRID:CVCL_3948), SNU398 (RRID:CVCL_0077), SNU423 (RRID:CVCL_0366), SNU449 (RRID:CVCL_0454), and SNU475 (RRID:CVCL_0497)) and other human cancer cell lines including lung cancer (A549 (RRID:CVCL_0023)), breast cancer (MCF‐7 (RRID:CVCL_0031)), and colon cancer (HCT‐116 (RRID:CVCL_0291)) were obtained from the Korean Cell Line Bank (KCLB, Seoul, South Korea). Cells were cultured in RPMI‐1640, Dulbecco's modified Eagle's medium (DMEM), or minimum essential medium (MEM) supplemented with 10% fetal bovine serum (FBS; Invitrogen, Carlsbad, CA, USA) and 1% penicillin–streptomycin (GenDEPOT), at 37 °C in a humidified incubator with 5% CO_2_. All cell lines were authenticated by short tandem repeat profiling and tested for mycoplasma contamination in February 2024 using the e‐Myco™ Mycoplasma PCR Detection Kit (ver. 2.0, LiliF, South Korea). All cell lines were confirmed to be mycoplasma‐free and were used within three years of authentication.

A *LUNAR* expression plasmid and corresponding empty control plasmid were designed and synthesized by VectorBuilder (Chicago, IL, USA); *LUNAR*, pRP[ncRNA]‐EGFP‐CMV>*LUNAR* (#VB221110‐1563exy) and control, pRP‐EGFP/Neo‐CAG>ORF_Stuffer (#VB010000‐9289pws). Small interfering RNA (siRNAs) and negative‐control RNA duplexes (siCtrl) were synthesized by Bioneer (Daejeon, South Korea) and Genolution (Seoul, South Korea), respectively. The sequences for siRNAs were as follows: non‐targeting scrambled sequence of siRNA (siNC), 5′‐UUCUCCGAACGUGUCACGUUU‐3′; siRNA targeting *TET1* (siTET1), 5′‐CGACCAAAACCUUGUGUGA‐3′; siRNA targeting *TET2* (siTET2), 5′‐ GAGGAAUAAAACGCACAGU‐3′. Cells were grown to 40% confluence; the transfection procedure was conducted using Lipofectamine 2000 transfection reagent (Invitrogen) according to the manufacturer's guidelines. After transfection, the cells were analyzed or processed in subsequent experiments.

### 2.6 Cell growth and viability

To analyze cell growth, HCC cells were seeded in 12‐well plates, transfected with *LUNAR* overexpression vector (EX‐*LUNAR*) or control vector (Empty vector). Subsequently, a solution of 3‐(4,5‐dimethylthiazol‐2‐yl)‐2,5‐diphenyltetrazolium bromide (MTT; Biosesang, Seongnam, South Korea) was added to each well every 24 h and incubated at 37 °C for 1 h. Formazan product can be dissolved in dimethyl sulfoxide, resulting in a purple color. Absorbance was measured at 570 nm using a TECAN SUNRISE Microplate Reader (TECAN, Zürich, Switzerland).

To analyze cell viability, HCC cells were transfected for 72 h in 60‐mm dishes. The cells were harvested by trypsinization, stained using 0.4% trypan blue solution (Invitrogen), and counted using a hemocytometer (Paul Marienfeld GmbH & Co. KG, Lauda‐Königshofen, Germany).

### 2.7 Wound healing assay

Cells were cultured and transfected with plasmid DNAs in 60‐mm dishes, reseeded into 6‐well plates at a cell density of about 100%, and finally incubated in a CO_2_ incubator at 37 °C. After overnight incubation, cell monolayers were scraped using a sterile 1000‐μL micropipette tip. Serum‐free medium was added to the cells after washing them with PBS to remove cell debris, and cells were incubated at 37 °C. Photographs of the gap widths were taken at 0 and 24 h, and images were obtained using an Olympus CKX53 microscope (Olympus, Tokyo, Japan) at 100× magnification. Each experiment was repeated three times and analyzed using ImageJ (version 1.49; Laboratory for Optical and Computational Instrumentation, Madison, WI, USA).

### 2.8 Transwell assay

For the transwell assay, 24‐well plates (Corning, New York, USA) and cell culture inserts (BD Biosciences, Franklin Lakes, New Jersey, USA) were used. The cell migration assay was performed without Matrigel, whereas the invasion assay was performed using Matrigel‐coated inserts. Matrigel (BD Biosciences) was diluted to a concentration of 0.3 mg·mL^−1^ in serum‐free media and 100 μL of the diluted Matrigel was added to the insert. HCC cells were cultured and transfected with plasmid DNAs for 24 h. Then, cells were reseeded in the upper insert (5 × 10^4^ cells) with serum‐free media and the lower cell culture insert was filled with 2.5–5% FBS‐containing media for 24–48 h at 37 °C in CO_2_ incubator. Non‐invading cells on the surface of the upper chamber were carefully removed. Migratory and invasive cells were stained using a Diff‐Quik staining kit (Sysmex Corporation, Chuo‐ku, Japan) and imaged using a CKX53 inverted microscope (Olympus, Tokyo, Japan) at 200× magnification. The experiments were independently repeated three times.

### 2.9 Sphere formation assay

Cells were seeded at a density of 3 × 10^3^ cells per well in 24‐well plates in culture medium containing an equal volume of Matrigel. After 10 days, spheres with diameters > 50 μm were imaged using a microscope.

### 2.10 Western blotting

Cell lysates were isolated using radio immunoprecipitation (RIPA) buffer containing Halt™ Protease Inhibitor Cocktail (Thermo Fisher Scientific, Waltham, MA, USA). Protein concentration was measured using a bicinchoninic acid (BCA) protein assay kit (Thermo Fisher Scientific), and protein lysates were resolved by sodium dodecyl sulfate polyacrylamide gel electrophoresis (SDS/PAGE) and transferred to polyvinylidene difluoride (PVDF) membranes (Merck Millipore, Burlington, MA, USA). The membranes were blocked for 1 h at room temperature (RT), incubated overnight with primary antibodies at 4 °C, and then incubated with horseradish peroxidase‐conjugated anti‐rabbit or anti‐mouse IgG for 1 h at RT. Chemiluminescence signals were detected with Clarity™ Western ECL Substrate (Bio‐Rad Laboratories) and visualized using ChemiDoc™ (Bio‐Rad Laboratories). The quantitative density of the bands was calculated using the ImageJ software. Antibody information is listed in Table S1.

### 2.11 Mouse orthotopic model

Six‐week‐old BALB/c female nude mice were obtained from ORIENT BIO (Seongnam, South Korea). Mice were housed in an individually ventilated cage system under specific pathogen‐free conditions, provided with food and water *ad libitum*, and acclimatized for one week before experimentation. We administered intraperitoneal injections of ketamine in Rompun solution (50 mg·kg^−1^ ketamine and 2.5 mg·kg^−1^ rompun) to induce anesthesia.

For the orthotopic xenograft assay, female BALB/c nude mice were randomly assigned to either the Empty vector group or the EX‐*LUNAR* group (*n* = 6 per group). Huh‐7 cells transfected with either Empty vector or EX‐*LUNAR* (5 × 10^5^ cells) were mixed with 50% Matrigel in serum‐free medium. Seven‐week‐old female BALB/c nude mice were anesthetized, and a small abdominal incision was made to expose the liver. The cell mixture was injected directly into the left hepatic lobe, after which the incision site was closed with surgical sutures. The mice were kept warm under a heat lamp until recovery from anesthesia. Nineteen days after cell injection, the mice were euthanized, and liver and tumor tissues were collected for hematoxylin and eosin (H&E) staining and immunohistochemistry (IHC) analyses.

For ethical considerations, the mice were euthanized at the endpoint of the experiments instead of being monitored until tumor‐related mortality. Euthanasia was performed by gradual‐fill carbon dioxide (CO_2_) inhalation in accordance with institutional animal care guidelines, and death was confirmed by cessation of respiration and heartbeat.

### 2.12 Immunohistochemistry (IHC)

IHC was used to detect the expression of protein markers. Mouse tumor tissues were harvested, fixed in 10% neutral buffered formalin, paraffin‐embedded, and cut into 5‐μm sections and then deparaffinized using xylene. For IHC, slices were hydrated in graded alcohol and then incubated overnight at 4 °C with primary antibodies. After washing thrice, the slices were incubated with secondary antibodies for 1 h, followed by incubation with a peroxidase substrate. Antibody information is listed in Table S1.

### 2.13 Gene set enrichment analysis (GSEA) and correlation analyses

To further investigate the relationship between *LUNAR* expression and NOTCH‐related transcriptional programs, Spearman correlation analyses were performed in the TCGA_LIHC cohort using a curated list of NOTCH pathway‐related genes. For pathway‐level analysis, TCGA_LIHC samples were divided into *LUNAR*‐high and *LUNAR*‐low groups according to the median expression value of *LUNAR*, and GSEA was performed using Hallmark gene sets.

In addition, an independent NOTCH intracellular domain (NICD)‐driven mouse HCC dataset (GSE33486) was analyzed. This dataset contains liver samples from control monotransgenic mice and HCC tissues from bitransgenic AFP‐Cre/Rosa26‐lsl‐NICD mice, in which the active NOTCH intracellular domain is constitutively expressed in hepatoblast‐derived cells. Differential expression analysis and GSEA were performed to assess the association between NOTCH activation and EMT‐related transcriptional programs.

### 2.14 TCGA methylation analysis

To evaluate the relationship between DNA methylation and *LUNAR* expression, correlation analyses were performed between *LUNAR* expression levels and beta values of CpG probes located within the *LUNAR* genomic region in the TCGA_LIHC dataset. CpG probes were selected based on annotation from the Illumina HumanMethylation450 platform. Correlation coefficients were calculated using Pearson's correlation analysis.

### 2.15 Quantitative methylation‐specific polymerase chain reaction (qMSP)

For the qMSP analysis, primers specific for either methylated or unmethylated DNA were designed using MethPrimer 2.0 (https://www.methprimer.com/cgi-bin/methprimer/methprimer.cgi). Briefly, bisulfite‐treated genomic DNA was amplified using amfiSure qGreen Q‐PCR Master Mix (GenDEPOT) under the following cycling conditions: 40 cycles of 95 °C for 15 s, 60 °C (for methylated primers) or 58 °C (for unmethylated primers) for 34 s, and 72 °C for 30 s, followed by a single cycle of 95 °C for 15 s, 60 °C for 60 s, and 95 °C for 15 s to generate dissociation curves. The reactions were monitored in real‐time using the CFX Connect Real‐Time PCR Detection System (Bio‐Rad Laboratories). The sequences of the primers used were as follows: *LUNAR*_Methyl: forward, 5′‐ ATTGGAGTTTGGTTAGTTGTTGG‐3′ and reverse, 5′‐ GCTAAAAATCACGAAATAAATACGAA‐3′; *LUNAR*_Unmethyl: forward, 5′‐TTGGAGTTTGGTTAGTTGTTGG‐3′ and reverse, 5′‐ACTAAAAATCACAAAATAAATACAAA‐3′. Relative DNA methylation was calculated using the difference between the Ct values of the methylated and unmethylated PCR products. All measurements were performed in triplicate.

### 2.16 5‐aza‐2′‐deoxycytidine (5‐Aza') treatment

Cells were treated with 8 μm 5‐Aza' (Sigma‐Aldrich, St. Louis, MO, USA) for 24–48 h at 37 °C in a CO_2_ incubator with the culture medium replaced daily. The treated cells were harvested and used to detect *LUNAR* expression.

### 2.17 Subcellular localization analysis

The nuclear and cytoplasmic fractions of MIHA and Huh‐7 cells were separated and purified using a PARIS Kit (Invitrogen) according to the manufacturer's instructions. Afterward, the expression levels of *LUNAR*, *GAPDH*, and *MALAT1* in the cytoplasm and nucleus of cells were examined by qRT‐PCR. *GAPDH* and *MALAT1* were used as controls of the cytoplasmic fraction and nuclear fraction, respectively.

### 2.18 Ethics statement

All experiments were performed in accordance with the Declaration of Helsinki and the study was approved by the Institutional Review Board of Ajou University Hospital (approval nos. AJIRB‐BMR‐KSP‐16‐365, AJIRB‐BMR‐SMP‐17‐189, AJOUIRB‐KSP‐2019‐417, AJOUIRB‐EX‐2022‐389, and AJOUIRB‐EX‐2024‐332). Anonymous serum samples and clinical data were provided by the Ajou Human Source Bank, and the requirement for informed consent was waived. All animals were cared for in accordance with the Guide for the Care and Use of Laboratory Animals, and the experiments were approved by the Ethics Committee of the Laboratory Animal Research Center of Ajou University Medical Center (IACUC_2022–0049), and animals were monitored daily for signs of distress or tumor burden. Humane endpoints were applied under the following conditions: (i) severe pain or stress associated with tumor growth, accompanied by > 20% body weight loss compared with pre‐tumor baseline; (ii) > 20% body weight loss compared with pre‐treatment baseline due to stress or adverse effects from oral anticancer drug administration; (iii) tumor‐associated ulceration or necrosis; (iv) tumor size exceeding 10% of the animal's normal body weight; (v) tumor volume approaching 1000 mm^3^, with euthanasia performed before exceeding this limit; and (vi) following tumor resection surgery, metastatic potential was monitored for up to 3 weeks, after which animals were euthanized.

### 2.19 Statistical analysis

All statistical analyses were performed using GraphPad Prism (version 10.0; GraphPad Software, San Diego, CA, USA) and R (version 4.2.3). Data are presented as mean ± standard deviation (SD) unless otherwise indicated. For comparisons between two groups, paired Student's *t*‐test was used for matched samples (e.g., T vs. adjacent NT tissues), and unpaired Welch's *t*‐test was applied for comparisons between independent groups (e.g., control vs. overexpression in cell lines). For multi‐group comparisons (e.g., NL, CH, LC, eHCC, aHCC), one‐way analysis of variance (ANOVA) with Tukey's post‐hoc test or the non‐parametric Kruskal–Wallis test was used as appropriate. Kaplan–Meier survival analysis was performed to evaluate differences in overall survival (OS) and disease‐specific survival (DSS) between high and low *LUNAR* expression groups, using the log‐rank test for statistical significance. Pearson correlation analysis was used to assess the association between methylation levels and *LUNAR* expression. Receiver operating characteristic (ROC) curves were generated to assess the diagnostic performance of *LUNAR* in four independent cohorts (TCGA_LIHC, GSE77314, GSE124535, and Ajou University cohort). The area under the curve (AUC), sensitivity, and specificity were calculated with 95% confidence intervals (CIs). All experiments were conducted in at least three biological replicates, and statistical significance was defined as *P* < 0.05.

## 3 Results

### 3.1 Identification of *LUNAR* as a liver‐specific lncRNA biomarker for HCC

**Fig. 1:**
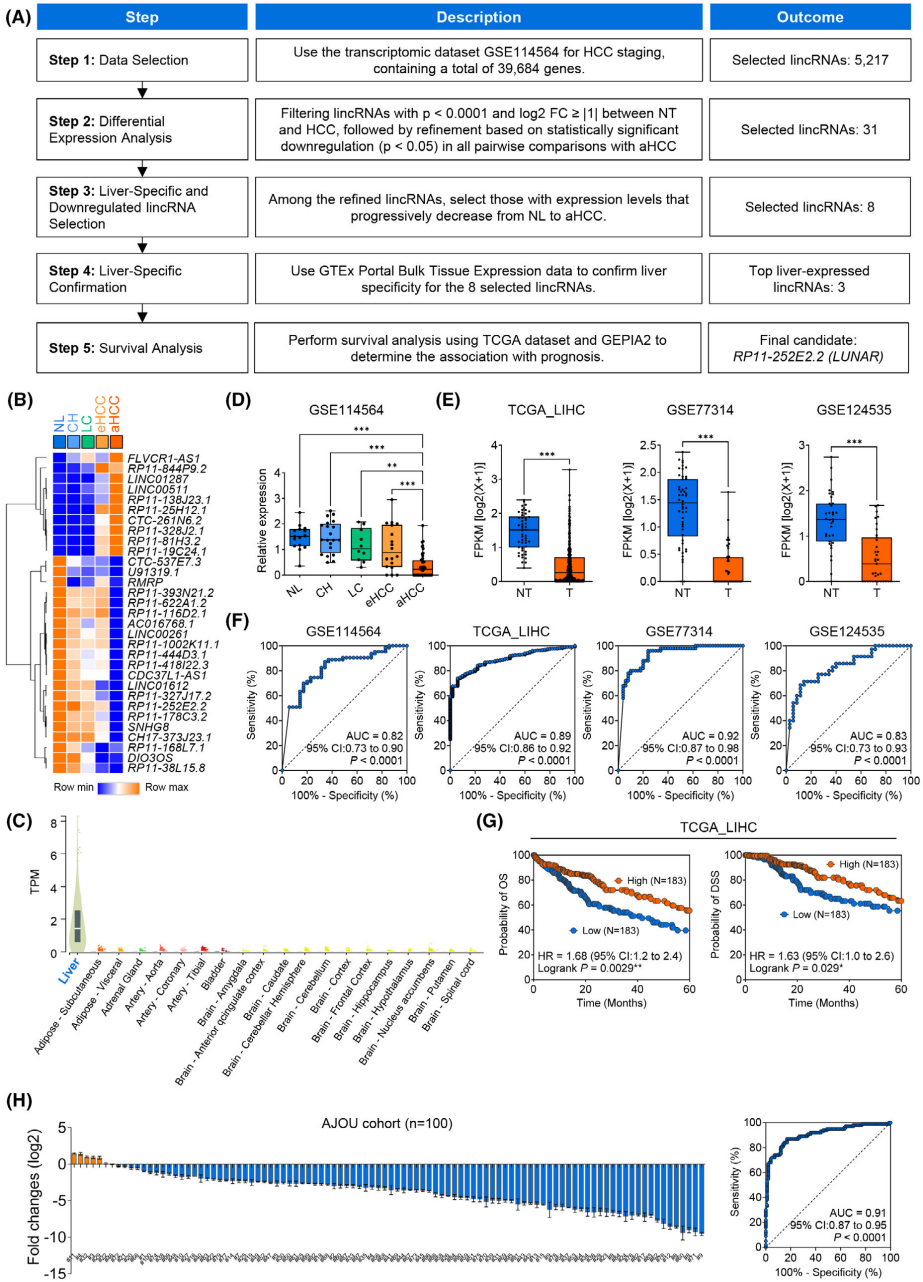
Identification and validation of *LUNAR* as a liver‐specific lncRNA. (A) Schematic workflow for the identification of candidate lncRNAs. (B) Heatmap visualization of the expression levels of 31 deregulated lincRNAs across different liver disease stages, based on the GSE114564 dataset. (C) Tissue‐specific expression profile of *RP11‐252E2.2* (*LUNAR*) obtained from the GTEx portal. (D) Analysis of *LUNAR* expression across normal liver (NL, *n* = 15), chronic hepatitis (CH, *n* = 20), liver cirrhosis (LC, *n* = 10), early HCC (eHCC, *n* = 18), and advanced HCC (aHCC, *n* = 45) samples from the GSE114564 dataset. (E) Comparative analysis of *LUNAR* expression in tumor (T) versus non‐tumor (NT) tissues from three public cohorts (TCGA_LIHC, NT [*n* = 50] vs. T [*n* = 371]; GSE77314, NT [*n* = 19] vs. T [*n* = 40]; GSE124535, NT [*n* = 6] vs. T [*n* = 70]). (F) ROC curve analysis to evaluate the diagnostic potential of *LUNAR* in four independent cohorts. (G) Kaplan–Meier survival analysis for overall survival (OS) and disease‐specific survival (DSS) based on *LUNAR* expression levels (high [*n* = 183] vs. low [*n* = 183]) in the TCGA_LIHC cohort; hazard ratios (HR) with 95% confidence intervals (CIs) and log‐rank P values are indicated. (H) qRT‐PCR validation of *LUNAR* expression in 100 paired tumor and NT tissues from the Ajou University Hospital cohort (left panel) and the corresponding ROC curve analysis (right panel). For the boxplots shown in panels D and E, the center line represents the median, the box represents the interquartile range (25th–75th percentile), and the whiskers indicate the minimum and maximum values. Data are presented as mean ± SD. Multi‐group comparisons were assessed by Kruskal–Wallis test; two‐group comparisons by unpaired Welch's *t*‐test or paired Student's *t*‐test as appropriate. Statistical significance levels (**P < 0.01, ***P < 0.001) are indicated where applicable.

To identify lncRNAs that specifically change as liver disease and liver cancer progress, we systematically analyzed transcriptomic profiles from the GSE114564 dataset, comprising NL, CH, LC, eHCC and aHCC (Fig. 1A). Starting from 39 684 genes, 5217 lncRNAs were identified as endogenously expressed at biologically meaningful levels, among which 21 were consistently downregulated and 10 were upregulated across all HCC stages compared to NL (Table S2). Among the 31 deregulated lncRNAs, 8 lncRNAs displayed a gradual decrease in expression across the disease spectrum from NL to aHCC (Fig. 1B).

To further prioritize tumor‐specific lncRNA candidates, we assessed liver specificity using bulk RNA‐seq data from the GTEx portal. Among the 8 lncRNAs that were both stage‐associated and downregulated, three lncRNAs, *CTC‐537E7.3*, *RP11‐252E2.2*, and *RP11‐38 L15.8*, exhibited liver‐specific expression profiles (Fig. 1C; Fig. S1). However, *RP11‐38 L15.8* showed no significant association with OS or DSS in the TCGA_LIHC cohort and was excluded from further analysis (data not shown). *CTC‐537E7.3*, although liver‐specific, had already been characterized in our previous study and published as a diagnostic marker [11]. Consequently, *RP11‐252E2.2* was selected as the final candidate based on its liver specificity, consistent stage‐dependent downregulation, and previously unreported clinical relevance. We therefore named this novel lncRNA *RP11‐252E2.2* as *LUNAR*, an acronym for Liver‐specific Upregulated Non‐coding RNA Down‐Regulator, as it is highly expressed in NL tissue and functions as a tumor suppressor in HCC. In the GSE114564 dataset, *LUNAR* expression showed a significant stepwise reduction across disease progression from NL to aHCC (*P* < 0.001, two‐way ANOVA test), supporting its potential as a progression‐related and repressed biomarker (Fig. 1D). To further validate this tendency, *LUNAR* expression was examined in three independent cohorts TCGA_LIHC, GSE77314, and GSE124535. In all datasets, *LUNAR* expression was significantly lower in T compared to matched NT tissues (*P* < 0.001, Fig. 1E).

Moreover, ROC curve analyses confirmed high diagnostic performance with AUC values of **0.84** (95% CI: 0.76–0.91) in GSE114564, 0.89 (95% CI: 0.86–0.92) in TCGA_LIHC, 0.92 (95% CI: 0.87–0.98) in GSE77314, and 0.83 (95% CI: 0.73–0.93) in GSE124535, respectively (Fig. 1F). Kaplan–Meier survival analyses of the TCGA_LIHC cohort demonstrated that patients with high *LUNAR* expression had significantly longer OS and DSS than those with low expression levels (Fig. 1G). Although the OS and DSS curves showed similar trends, DSS specifically reflects HCC‐related mortality, indicating that the prognostic impact of *LUNAR* is directly associated with liver cancer outcomes rather than overall patient survival alone. Finally, to validate these findings in an independent clinical cohort, we performed qRT‐PCR on paired T and adjacent NT liver tissues from 100 HCC patients at Ajou University Hospital. *LUNAR* expression was significantly decreased in liver cancer tissues (*P* < 0.0001; left panel), and ROC analysis yielded an AUC of 0.91 (95% CI: 0.87–0.95; right panel), consistent with public datasets (Fig. 1H). To further evaluate the clinical relevance of *LUNAR* in a cirrhotic background, we performed additional subgroup analyses using the GSE114564 dataset and independent extracellular vesicle (EV) cohorts. *LUNAR* expression was significantly lower in HCC compared with LC; however, no significant difference was observed between LC and eHCC (Fig. S2A,B).

To assess its potential as a non‐invasive biomarker, we further analyzed EV‐derived *LUNAR* expression using both the exoRBase 3.0 database and an independent Ajou University Hospital cohort. Although *LUNAR* was detectable in circulating EVs, no significant differences were observed between disease groups (Fig. S2C,D).

Taken together, these findings indicate that although *LUNAR* expression progressively declines during hepatocarcinogenesis, its ability to discriminate LC from eHCC, particularly in a circulating EV‐based setting, is limited. These results suggest that *LUNAR* is more closely associated with disease progression rather than early tumor detection, supporting its role as a tissue‐associated biomarker in HCC.

### 3.2 Functional analysis of *LUNAR* reveals its inhibitory role in HCC cell motility

To characterize its functional role in HCC, we examined endogenous *LUNAR* expression and found that it was highly expressed in the normal hepatocyte line MIHA but markedly downregulated in all eight tested HCC cell lines (Hep3B, Huh‐7, PLC/PRF/5, SNU368, SNU398, SNU423, SNU449, and SNU475) (Fig. S3A). We next transfected the *LUNAR* overexpression vector (EX‐*LUNAR*) into five representative HCC cell lines (Hep3B, Huh‐7, PLC/PRF/5, SNU449, and SNU475) and confirmed its robust expression (Fig. S3B). Importantly, vector‐mediated overexpression of *LUNAR* had no significant impact on cell proliferation as confirmed by cell counting (Fig. S3C).

**Fig. 2:**
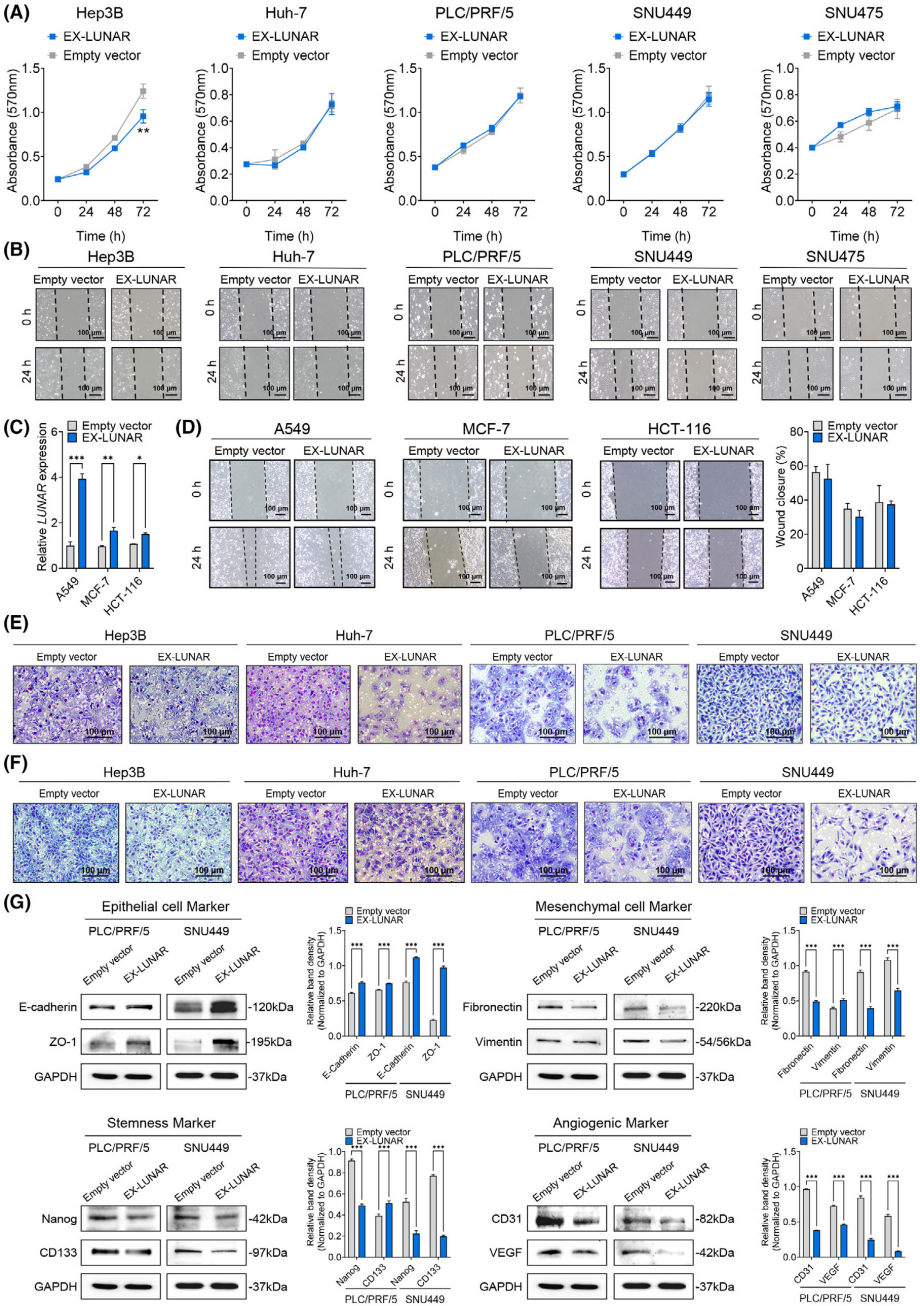
Functional assays to determine the role of *LUNAR* in HCC. (A) Cell viability assessment by MTT assay in five HCC cell lines (Hep3B, Huh‐7, PLC/PRF/5, SNU449, and SNU475) transfected with an empty vector or a *LUNAR* overexpression vector (EX‐*LUNAR*) over 72 h. (B) Wound healing assay performed to assess cell migration in five HCC cell lines (Hep3B, Huh‐7, PLC/PRF/5, SNU449, and SNU475) transfected with an empty vector or EX‐*LUNAR*; images were taken at 0 and 24 h. (C) qRT‐PCR confirmation of EX‐*LUNAR* expression in non‐hepatic cancer cell lines (A549, MCF‐7, and HCT‐116). (D) Comparative wound healing assays in non‐hepatic cancer cell lines (A549, MCF‐7, and HCT‐116) transfected with an empty vector or EX‐*LUNAR*; images were taken at 0 and 24 h. (E) Transwell migration assay with four HCC cell lines (Hep3B, Huh‐7, PLC/PRF/5, and SNU449) transfected with an empty vector or EX‐*LUNAR*. (F) Transwell invasion assay using Matrigel‐coated inserts with four HCC cell lines (Hep3B, Huh‐7, PLC/PRF/5, and SNU449) transfected with an empty vector or EX‐*LUNAR*. (G) Western blot analysis for the expression of epithelial, mesenchymal, stemness, and angiogenic markers in PLC/PRF/5 and SNU449 cells following transfection with an empty vector or EX‐*LUNAR*; representative blots and quantified band densities normalized to GAPDH are shown. All data are presented as mean ± SD from at least three independent experiments. Statistical comparisons were performed using unpaired Welch's *t*‐test. Statistical significance levels (*P < 0.05, **P < 0.01, ***P < 0.001) are indicated where applicable.

Consistent with these findings, MTT assays performed over 72 h showed no significant change in cell viability upon *LUNAR* overexpression in the five cell lines (Fig. 2A), except for a modest but statistically significant decrease observed at 72 h in Hep3B cells. This suggests that *LUNAR* does not exert cytotoxic effects under standard culture conditions. We next assessed the effect of *LUNAR* on cell motility. Wound healing assays demonstrated a significant reduction in migration upon *LUNAR* overexpression in all five HCC cell lines (Fig. 2B; Fig. S3D). To determine whether this anti‐migratory effect was liver cancer‐specific, we confirmed robust *LUNAR* overexpression in non‐hepatic cancer cell lines, including A549, MCF‐7, and HCT‐116 (Fig. 2C). In contrast to HCC cells, *LUNAR* overexpression did not significantly suppress migration in these non‐hepatic cancer cell lines, suggesting that the inhibitory effect of *LUNAR* on cell motility is context‐dependent and preferentially observed in HCC cells (Fig. 2D). In transwell migration assays, *LUNAR* significantly suppressed the migratory capacity of Hep3B, Huh‐7, PLC/PRF/5, and SNU449 cells (Fig. 2E; Fig. S3E). Similarly, invasion assays revealed a pronounced reduction in invasive ability in the same four HCC cell lines upon *LUNAR* overexpression (Fig. 2F; Fig. S3F).

To explore the mechanism underlying these phenotypic changes, we performed western blot analysis targeting major EMT‐related markers. *LUNAR* overexpression resulted in increased expression of epithelial markers, alongside decreased levels of mesenchymal markers, indicating a reversal of the EMT process (Fig. 2G, upper panels). Furthermore, markers associated with stemness and angiogenesis were also downregulated (Fig. 2G, lower panels), suggesting that *LUNAR* suppresses HCC cell aggressiveness through multifaceted regulation of cellular plasticity and invasive traits.

### 3.3 *LUNAR* suppresses stemness‐associated tumorsphere formation in HCC cells

**Fig. 3:**
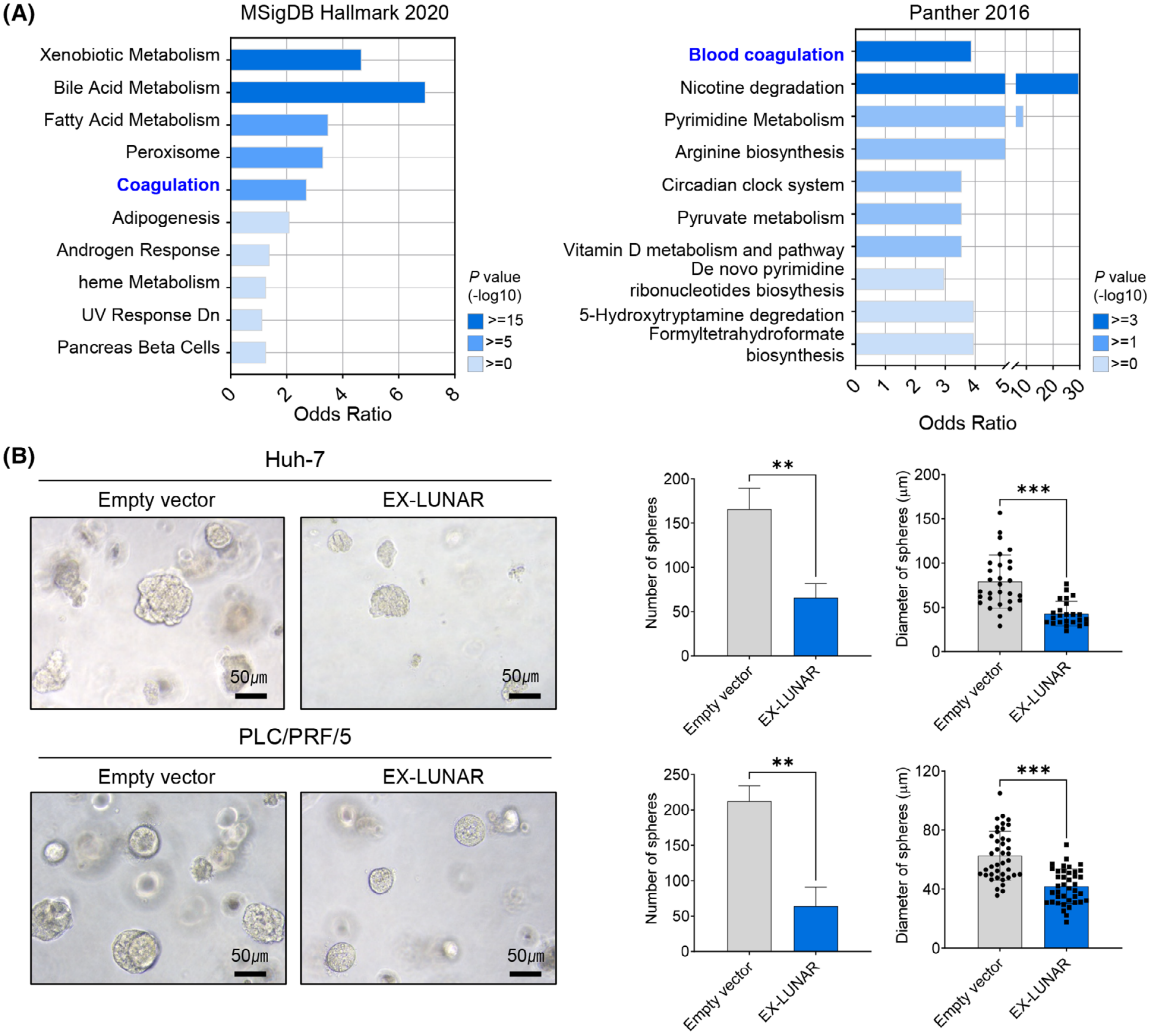
Pathway enrichment and stemness analysis of *LUNAR* in HCC. (A) Gene set enrichment analysis (GSEA) of pathways correlated with *LUNAR* expression using MSigDB Hallmark 2020 and Panther 2016 databases. Genes exhibiting a Pearson correlation coefficient > 0.2 with *LUNAR* expression in the TCGA_LIHC dataset were subjected to pathway enrichment analysis. (B) Tumorsphere formation assay in Huh‐7 and PLC/PRF/5 cells transfected with an empty vector or EX‐*LUNAR*. Representative images are shown at ×200 magnification (scale bar = 50 μm). The number and diameter of tumorspheres were quantified (*n* = 3 independent experiments per group). For tumorsphere diameter analysis, each dot represents an individual tumorsphere. Data are presented as mean ± SD. Statistical comparisons were performed using unpaired Welch's *t*‐test. Statistical significance levels (**P < 0.01, ***P < 0.001) are indicated where applicable.

To further explore biological programs associated with *LUNAR* expression, we performed pathway enrichment analysis using genes positively correlated with *LUNAR* expression in the TCGA_LIHC cohort. Genes exhibiting a Pearson correlation coefficient greater than 0.2 with *LUNAR* were subjected to enrichment analysis using the MSigDB Hallmark 2020 and Panther 2016 databases. This analysis identified metabolic and liver‐associated pathways, including xenobiotic metabolism, bile acid metabolism, fatty acid metabolism, peroxisome, and coagulation‐related pathways, as being enriched among *LUNAR*‐associated genes (Fig. 3A), suggesting that *LUNAR* expression is linked to liver‐enriched transcriptional programs and metabolic features that are progressively lost during hepatocarcinogenesis.

Given the observed effects of *LUNAR* on EMT, migration, invasion, and metastasis‐related phenotypes, we next examined whether *LUNAR* also affects stemness‐associated properties of HCC cells. Tumorsphere formation assays were performed in Huh‐7 and PLC/PRF/5 cells transfected with an empty vector or EX‐*LUNAR*. *LUNAR* overexpression markedly reduced tumorsphere formation in both cell lines, as shown by decreases in both the number and diameter of tumorspheres (Fig. 3B).

Collectively, these findings provide additional functional evidence that LUNAR restrains metastatic plasticity in HCC, including not only migratory and invasive traits but also stemness‐associated tumor cell properties.

### 3.4 Anti‐metastatic function of *LUNAR in vivo*

**Fig. 4:**
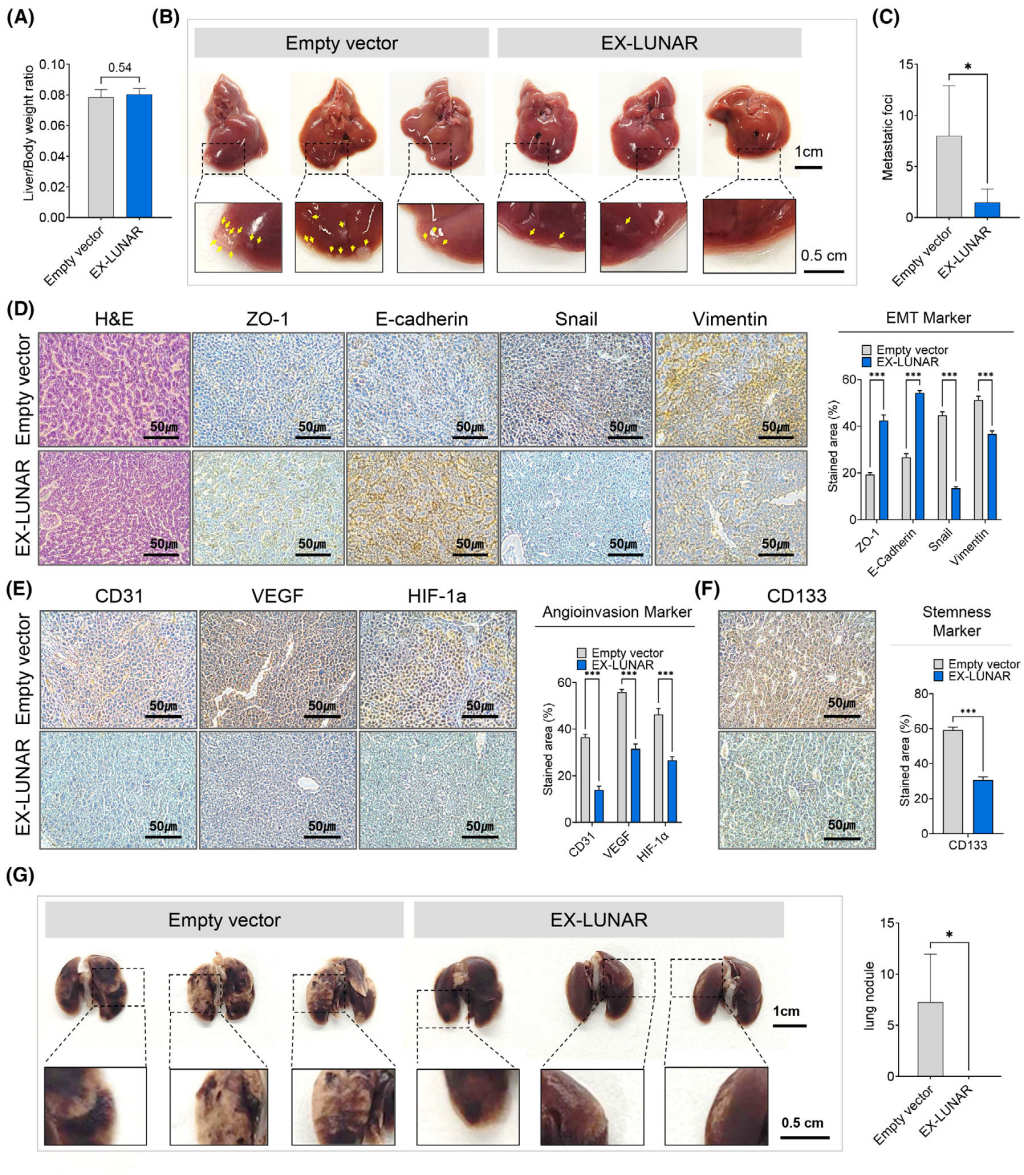
*In vivo* analysis of *LUNAR* function in an orthotopic mouse model. (A) Liver‐to‐body weight ratios in mice injected with Huh‐7 cells transfected with an empty vector or EX‐*LUNAR* (n = 6 per group). (B) Representative macroscopic images of livers 19 days post‐injection. Three representative liver images per group are shown. Yellow arrows indicate intrahepatic tumor nodules. Scale bars = 1 cm (upper) and 0.5 cm (lower). (C) Quantification of intrahepatic metastatic foci in the empty vector and EX‐*LUNAR* groups (*n* = 6 per group). (D) Representative H&E staining and IHC staining of EMT‐related markers, including ZO‐1, E‐cadherin, Snail, and Vimentin, in orthotopic tumor tissues, with quantification of relative staining intensity shown on the right. Scale bar = 50 μm. (E) Representative IHC staining of angiogenic markers, including CD31, VEGF, and HIF‐1α, in orthotopic tumor tissues, with quantification of relative staining intensity shown on the right. Scale bar = 50 μm. (F) Representative IHC staining of the stemness marker CD133 in orthotopic tumor tissues, with quantification of relative staining intensity shown on the right. Scale bar = 50 μm. (G) Representative macroscopic images of lungs and quantification of spontaneous lung metastatic nodules in the empty vector and EX‐*LUNAR* groups (*n* = 6 per group). Scale bars = 1 cm (upper) and 0.5 cm (lower). Data are presented as mean ± SD. Statistical comparisons were performed using unpaired Welch's *t*‐test. Statistical significance levels (*P < 0.05, ***P < 0.001) are indicated where applicable.

To evaluate the functional relevance of *LUNAR in vivo*, we first assessed its biosafety profile using an orthotopic liver cancer mouse model. As shown in Fig. 4A, no significant differences were observed in liver‐to‐body weight ratios between mice injected with control (empty vector) or Huh‐7 EX‐*LUNAR* cells, indicating that *LUNAR* overexpression does not cause overt systemic toxicity. We next examined whether *LUNAR* suppresses intrahepatic tumor growth and metastasis. In orthotopic xenograft models, intrahepatic tumor foci were prominently observed in mice injected with control cells, whereas EX‐*LUNAR* mice showed markedly fewer and smaller tumor nodules (Fig. 4B). Quantification confirmed a statistically significant reduction in intrahepatic metastatic foci in the EX‐*LUNAR* group (Fig. 4C), suggesting that *LUNAR* suppresses local tumor progression and intrahepatic dissemination. Given the strong *in vitro* evidence implicating *LUNAR* in EMT regulation, we further examined the tumors histologically. H&E staining combined with immunohistochemistry revealed that tumors from EX‐*LUNAR* mice exhibited increased expression of epithelial markers E‐cadherin and ZO‐1, and decreased expression of mesenchymal markers Vimentin and Snail (Fig. 4D). Moreover, angiogenesis‐related markers such as CD31 and VEGF (Fig. 4E), and stemness markers including CD133 (Fig. 4F), were also markedly reduced in *LUNAR*‐overexpressing tumors. In line with our *in vitro* findings, *LUNAR* overexpression did not significantly alter tumor cell proliferation *in vivo*, as evidenced by comparable expression levels of Ki‐67 and DNA‐PKs in orthotopic tumors (Fig. S4). We next examined spontaneous lung metastases in the orthotopic xenograft model. Notably, lung metastatic nodules were frequently observed in the control group but significantly reduced in the EX‐*LUNAR* group, supporting the anti‐metastatic role of *LUNAR in vivo* (Fig. 4G). Together, these *in vivo* findings demonstrate that *LUNAR* robustly suppresses intrahepatic and systemic dissemination of HCC cells while modulating key oncogenic pathways related to EMT, angiogenesis, and cancer stemness without inducing toxicity.

### 3.5 *LUNAR* is epigenetically repressed by DNA hypermethylation

Although *RP11‐252E2.2* was previously reported as a hypermethylated transcript, its functional epigenetic regulation had not been fully elucidated. Based on this prior evidence and our observation of liver‐specific downregulation of *LUNAR*, we hypothesized that promoter hypermethylation contributes to its transcriptional repression in HCC [12].

**Fig. 5:**
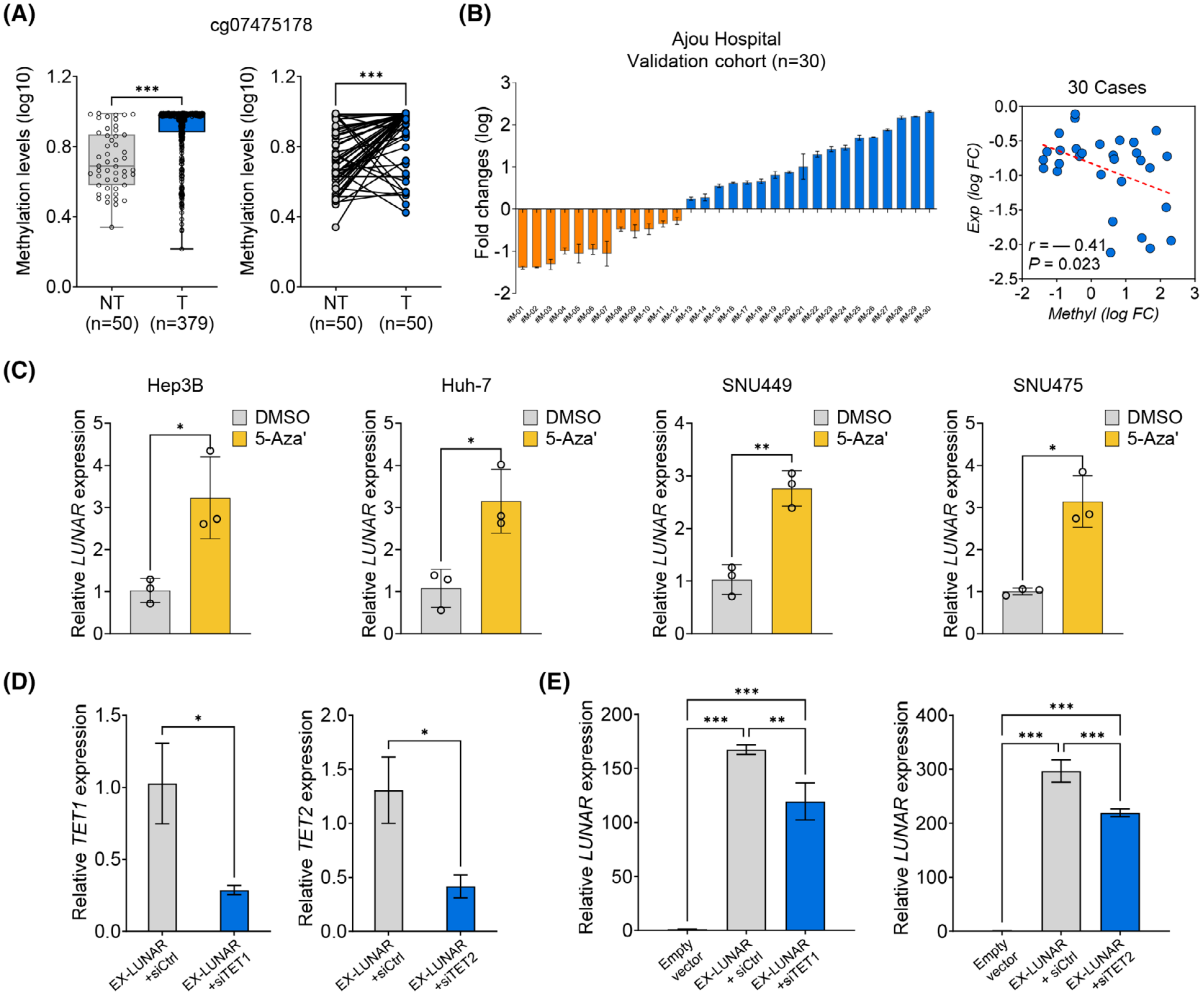
Investigation of the epigenetic regulation of *LUNAR*. (A) Analysis of methylation levels at CpG site cg07475178 in non‐tumor (NT, *n* = 50) and tumor (T, *n* = 379) tissues from the TCGA_LIHC dataset (left), and in paired NT (*n* = 50) and T (*n* = 50) clinical samples (right). (B) Quantitative methylation‐specific PCR (qMSP) in a validation cohort of 30 paired T and NT tissues from Ajou University Hospital (left), and Pearson correlation analysis between methylation levels and *LUNAR* expression (right; *r* = −0.41, *n* = 30). (C) qRT‐PCR analysis of *LUNAR* expression in four HCC cell lines (Hep3B, Huh‐7, SNU449, and SNU475) following treatment with 5‐aza‐2′‐deoxycytidine (5‐Aza', 8 μm) or DMSO control for 24–48 h. (D) qRT‐PCR assessment of *TET1* and *TET2* expression following siRNA‐mediated knockdown in Huh‐7 cells. (E) qRT‐PCR analysis of *LUNAR* expression in Huh‐7 cells after co‐transfection with EX‐*LUNAR* and siRNAs targeting *TET1* or *TET2*. For the boxplot shown in panel A, the center line represents the median, the box represents the interquartile range (25th–75th percentile), and the whiskers indicate the minimum and maximum values. Data are presented as mean ± SD. Statistical comparisons were performed using paired Student's *t*‐test for paired tissue samples and unpaired Welch's *t*‐test for independent group comparisons. Pearson correlation analysis was used for correlation testing. All experiments were independently repeated three times. Statistical significance levels (*P < 0.05, **P < 0.01, ***P < 0.001) are indicated where applicable.

To investigate this, we analyzed TCGA methylation array data and identified twelve CpG probes located at the *LUNAR* genomic locus. Among them, only the CpG site cg07475178 showed significantly higher methylation in T than in adjacent NT tissues in the TCGA_LIHC samples and was therefore selected for further study (Fig. 5A; Fig. S5A). To further determine whether *LUNAR* expression is broadly associated with methylation across its genomic region, we performed correlation analyses between *LUNAR* expression and all twelve CpG probes. Several CpG sites exhibited inverse correlations with *LUNAR* expression, indicating a general negative association between DNA methylation and *LUNAR* expression. Notably, cg07475178 showed the strongest inverse correlation among these sites (*r* = −0.26, *P* < 0.001), supporting its potential functional relevance (Fig. S5B). To validate this observation, we conducted qMSP on 30 paired T and NT liver tissues collected from the Ajou University Hospital cohort using primers designed with MethPrimer (Fig. S5C). Consistent with the TCGA data, most T samples exhibited increased methylation levels compared to matched NT tissues (Fig. 5B, left). Furthermore, methylation level negatively correlated with *LUNAR* expression (*r* = −0.41), suggesting that promoter hypermethylation contributes to its transcriptional silencing in HCC (Fig. 5B, right). To further verify whether DNA methylation mediates the suppression of *LUNAR*, we treated HCC cells with 5‐Aza'. *LUNAR* expression was significantly restored following treatment, indicating that DNA methylation contributes to *LUNAR* suppression (Fig. 5C). To explore whether active demethylation pathways also influence *LUNAR* regulation, we repressed *TET1* and *TET2* in Huh‐7 cells using siRNA. The knockdown efficiency of *TET1* and *TET2* was confirmed by qRT‐PCR in Huh‐7 cells overexpressing *LUNAR*, showing a significant reduction in mRNA levels compared to controls (Fig. 5D), and subsequent measurement of *LUNAR* expression showed that loss of either *TET1* or *TET2* significantly reduced *LUNAR* transcript levels (Fig. 5E). These findings are consistent with a role for TET‐mediated demethylation in maintaining *LUNAR* expression, although direct binding of TET1 or TET2 to the *LUNAR* promoter has not yet been demonstrated. Collectively, these results suggest that *LUNAR* expression may be influenced by DNA methylation and TET‐mediated demethylation, underscoring its epigenetic vulnerability and highlighting it as a potential target for epigenetic therapy in HCC.

### 3.6 *LUNAR* is predominantly nuclear and preferentially attenuates NOTCH pathway components in HCC

**Fig. 6:**
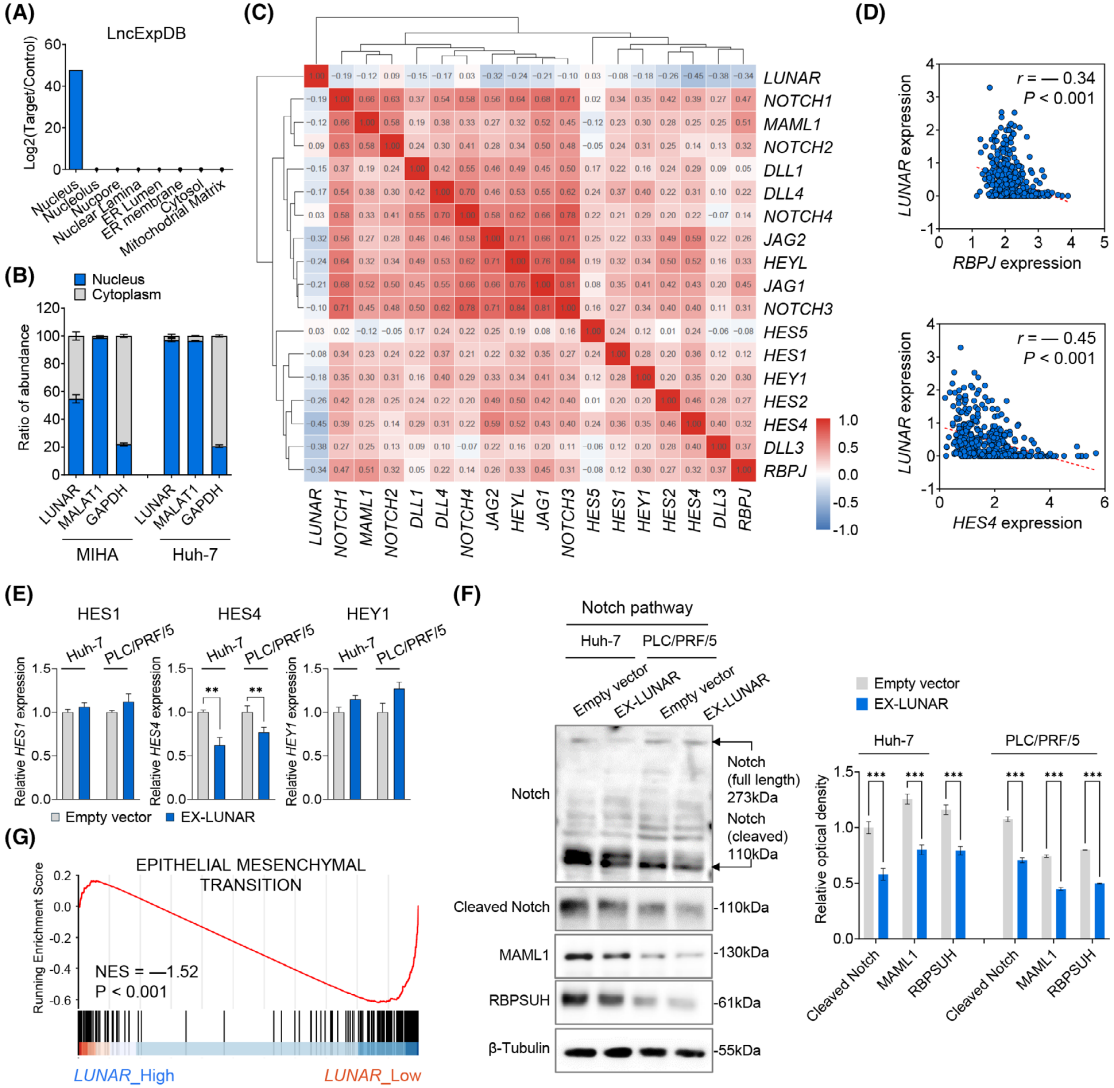
Subcellular localization of *LUNAR* and its association with NOTCH pathway activity in HCC. (A) Subcellular localization of *LUNAR* based on publicly available data from LncExpDB, showing predominant nuclear enrichment in HEK293T cells. (B) Nuclear–cytoplasmic fractionation assay in NT hepatocytes (MIHA) and HCC cells (Huh‐7). *LUNAR* expression was quantified in nuclear and cytoplasmic fractions by qRT‐PCR. *GAPDH* and *MALAT1* were used as cytoplasmic and nuclear markers, respectively, to confirm fractionation purity. (C) Heatmap showing Spearman correlation coefficients between *LUNAR* expression and NOTCH pathway‐related genes in the TCGA_LIHC cohort. (D) Representative scatter plots showing inverse correlations between *LUNAR* expression and *RBPJ* (*r* = −0.34, *P* < 0.001) and *HES4* (*r* = −0.45, *P* < 0.001) in the TCGA_LIHC cohort. (E) qRT‐PCR analysis of canonical NOTCH target genes (*HES1*, *HES4*, and *HEY1*) in Huh‐7 and PLC/PRF/5 cells transfected with an empty vector or EX‐*LUNAR*. (F) Western blot analysis of NOTCH signaling pathway components (Notch full length, Cleaved Notch, MAML1, and RBPSUH) in Huh‐7 and PLC/PRF/5 cells transfected with an empty vector or EX‐*LUNAR*; representative blots and quantified band densities normalized to β‐Tubulin are shown. (G) Gene set enrichment analysis (GSEA) showing negative enrichment of the epithelial–mesenchymal transition (EMT) gene set in *LUNAR*‐high versus *LUNAR*‐low tumors in the TCGA_LIHC cohort (normalized enrichment score [NES] = −1.52, *P* < 0.001). Data are presented as mean ± SD (*n* = 3 independent experiments per group). Statistical comparisons were performed using unpaired Welch's *t*‐test. Statistical significance levels (**P < 0.01, ***P < 0.001) are indicated where applicable.

To gain mechanistic insight into how *LUNAR* regulates HCC progression, we first examined its subcellular localization. Publicly available LncExpDB data showed predominant nuclear enrichment of *LUNAR* in HEK293T cells (Fig. 6A). Consistently, nuclear–cytoplasmic fractionation assays in MIHA and Huh‐7 cells demonstrated that *LUNAR* was mainly localized in the nuclear fraction, with stronger nuclear enrichment in Huh‐7 cells. *GAPDH* and *MALAT1* were used as cytoplasmic and nuclear markers, respectively, to confirm fractionation purity (Fig. 6B).

We next assessed the association between *LUNAR* and NOTCH pathway activity. Spearman correlation analysis in the TCGA_LIHC cohort showed that *LUNAR* expression was inversely correlated with multiple NOTCH pathway‐related genes (Fig. 6C). Representative scatter plots further confirmed significant inverse correlations between *LUNAR* and *RBPJ* (*r* = −0.34, *P* < 0.001) and between *LUNAR* and *HES4* (*r* = −0.45, *P* < 0.001) (Fig. 6D). At the cellular level, qRT‐PCR analysis showed that *LUNAR* overexpression significantly reduced *HES4* expression in both Huh‐7 and PLC/PRF/5 cells, whereas *HES1* and *HEY1* did not show consistent changes (Fig. 6E). Western blot analysis further demonstrated that *LUNAR* overexpression reduced cleaved NOTCH, MAML1, and RBPSUH protein levels in both Huh‐7 and PLC/PRF/5 cells (Fig. 6F). By contrast, JAK/STAT and ERK/AKT signaling components were not markedly altered by *LUNAR* overexpression (Fig. S6A,B), supporting a relatively selective association between *LUNAR* and NOTCH pathway attenuation. In addition, GSEA revealed negative enrichment of EMT‐related gene sets in *LUNAR*‐high tumors compared with *LUNAR*‐low tumors in the TCGA_LIHC cohort (normalized enrichment score [NES] = −1.52, *P* < 0.001) (Fig. 6G). Conversely, EMT‐related gene sets and representative EMT‐associated genes were enriched in an independent NICD‐driven mouse HCC dataset (Fig. S6C,D), supporting the link between NOTCH activation and EMT‐associated transcriptional programs.

Together, these findings indicate that LUNAR is predominantly nuclear and is associated with preferential attenuation of NOTCH pathway components and EMT‐related transcriptional activity in HCC.

## 4 Discussion

Here, we describe *LUNAR*, a previously uncharacterized lncRNA that is highly expressed in NL but progressively downregulated across LC, eHCC, and advanced disease. Multi‐cohort analysis confirmed a stepwise decline in *LUNAR*, with AUC values up to 0.92 for distinguishing T from NT tissue. Low *LUNAR* levels were significantly associated with shorter overall survival and disease‐specific survival, underscoring its diagnostic and prognostic relevance in HCC.

Functionally, *LUNAR* expression suppressed migration and invasion in HCC cells without altering proliferation, and curtailed intrahepatic and pulmonary metastasis in an orthotopic mouse model, all without systemic toxicity. *LUNAR* re‐established epithelial identity and downregulated angiogenic and stemness markers, suggesting a broad inhibitory effect on metastatic plasticity. Moreover, *LUNAR* is a nucleus‐enriched lncRNA that preferentially attenuates the NOTCH pathway, leaving JAK/STAT and ERK/AKT signaling unaffected. In the TCGA_LIHC cohort, *LUNAR* expression was inversely correlated with several NOTCH pathway‐related genes, including *RBPJ* and *HES4*. At the cellular level, *LUNAR* overexpression selectively reduced *HES4* expression, whereas *HES1* and *HEY1* did not show consistent changes, suggesting preferential attenuation of selected NOTCH‐associated transcriptional components rather than broad suppression of all canonical NOTCH targets. Given the established role of NOTCH signaling in EMT, stemness, and therapy resistance, its attenuation may contribute to the anti‐metastatic phenotype [13, 14, 15].

While our data indicate that *LUNAR* overexpression is associated with attenuation of NOTCH signaling, the extent to which the observed anti‐migratory and anti‐invasive effects are dependent on NOTCH activity remains to be fully determined. It is therefore possible that additional NOTCH‐independent mechanisms also contribute to the functional effects of *LUNAR*. Furthermore, *LUNAR* appears to be epigenetically regulated by promoter hypermethylation. A key CpG probe (cg07475178) displayed tumor‐restricted hypermethylation inversely correlated with transcript abundance; 5‐Aza restored *LUNAR* in HCC cells. Knockdown of the demethylases *TET1/2* likewise reduced *LUNAR*, suggesting that both increased methylation and reduced active demethylation may contribute to *LUNAR* downregulation, although the direct binding of *TET1* or *TET2* to the *LUNAR* promoter remains to be experimentally demonstrated.

Likewise, several limitations of the present study warrant acknowledgment. The precise molecular mechanism by which *LUNAR* modulates NOTCH pathway activity remains to be fully elucidated, and rescue experiments using constitutively active NOTCH were beyond the scope of the current study. Mapping *LUNAR*'s protein and chromatin partners through high‐throughput interactome approaches will be essential to clarify its mode of action. Although *LUNAR* showed robust tissue‐level downregulation, its ability to distinguish LC from eHCC and its discriminatory performance in circulating EVs were limited in the current datasets. Thus, *LUNAR* should be interpreted primarily as a tissue‐associated and progression‐associated biomarker candidate rather than as a standalone marker for early non‐invasive detection. Because we evaluated the cell‐autonomous roles of *LUNAR* using an athymic nude mouse model, its impact on the tumor immune microenvironment—such as via PD‐L1 expression or chemokine regulation—remains unexplored. Future studies using immunocompetent syngeneic models will be essential to define whether *LUNAR* modulates anti‐tumor immunity and whether it can synergize with immune checkpoint inhibitors. Finally, validation across diverse etiologies and ethnic cohorts, together with comprehensive long‐term safety studies, will be necessary before clinical translation can be considered.

## 5 Conclusion

In summary, *LUNAR* is a liver‐enriched lncRNA epigenetically downregulated during hepatocarcinogenesis, and its restoration restrains metastatic progression while being associated with preferential attenuation of NOTCH pathway components. Its tissue‐specific expression and functional effects highlight its potential as a tissue‐associated biomarker candidate and therapeutic target in HCC.

## Conflict of interest

The authors declare no conflict of interest.

## Author contributions

JYC: conceptualization, writing – original draft, funding acquisition, supervision. JWE: conceptualization, investigation, writing – review & editing, visualization, funding acquisition, supervision. SHJ: methodology, investigation, writing – original draft, visualization. HSK: methodology, investigation, writing – review & editing, funding acquisition. GOB: methodology, validation, revision. SIL: methodology, validation. JYJ: methodology. MGY: investigation, validation. SSK: funding acquisition, investigation. JEH: data curation, editing.

## Supporting information


**Fig. S1.** Tissue‐specific expression profiles of candidate lncRNAs.
**Fig. S2.** Evaluation of *LUNAR* expression in cirrhosis versus HCC and in circulating extracellular vesicles.
**Fig. S3.** Quantitative analysis of *in vitro* functional assays.
**Fig. S4.** Effect of *LUNAR* overexpression on tumor cell proliferation *in vivo*.
**Fig. S5.** Supporting data for the epigenetic regulation of *LUNAR*.
**Fig. S6.** Supporting analyses of JAK/STAT, ERK/AKT, and NOTCH‐associated EMT signaling.
**Table S1.** Antibodies used for western blot and Immunohistochemistry.
**Table S2.** Differentially expressed lncRNAs identified across liver disease progression stages in GSE114564.

## Data Availability

The data supporting the findings of this study are available from the corresponding author upon reasonable request.
