# Supplementary material for: Epigenetic silencing of the liver‐specific lncRNA LUNAR promotes liver cancer progression via NOTCH activation
Source: Mol Oncol. 2026 Jul 12:10.1002/1878-0261.70301. Online ahead of print. doi: 10.1002/1878-0261.70301 (PMC13399082; doi:10.1002/1878-0261.70301)
Supplement: Supplementary file 1 — Fig. S1. Tissue‐specific expression profiles of candidate lncRNAs. Fig. S2. Evaluation of LUNAR expression in cirrhosis versus HCC and in circulating extracellular vesicles. Fig. S3. Quantitative analysis of in vitro functional assays. Fig. S4. Effect of LUNAR overexpression on tumor cell proliferation in vivo. Fig. S5. Supporting data for the epigenetic regulation of LUNAR. Fig. S6. Supporting analyses of JAK/STAT, ERK/AKT, and NOTCH‐associated EMT signaling. Table S1. Antibodies used for western blot and Immunohistochemistry. Table S2. Differentially expressed lncRNAs identified across liver disease progression stages in GSE114564. [file MOL2-9999-0-s001.docx]

Supplementary Information

Supplementary Figures


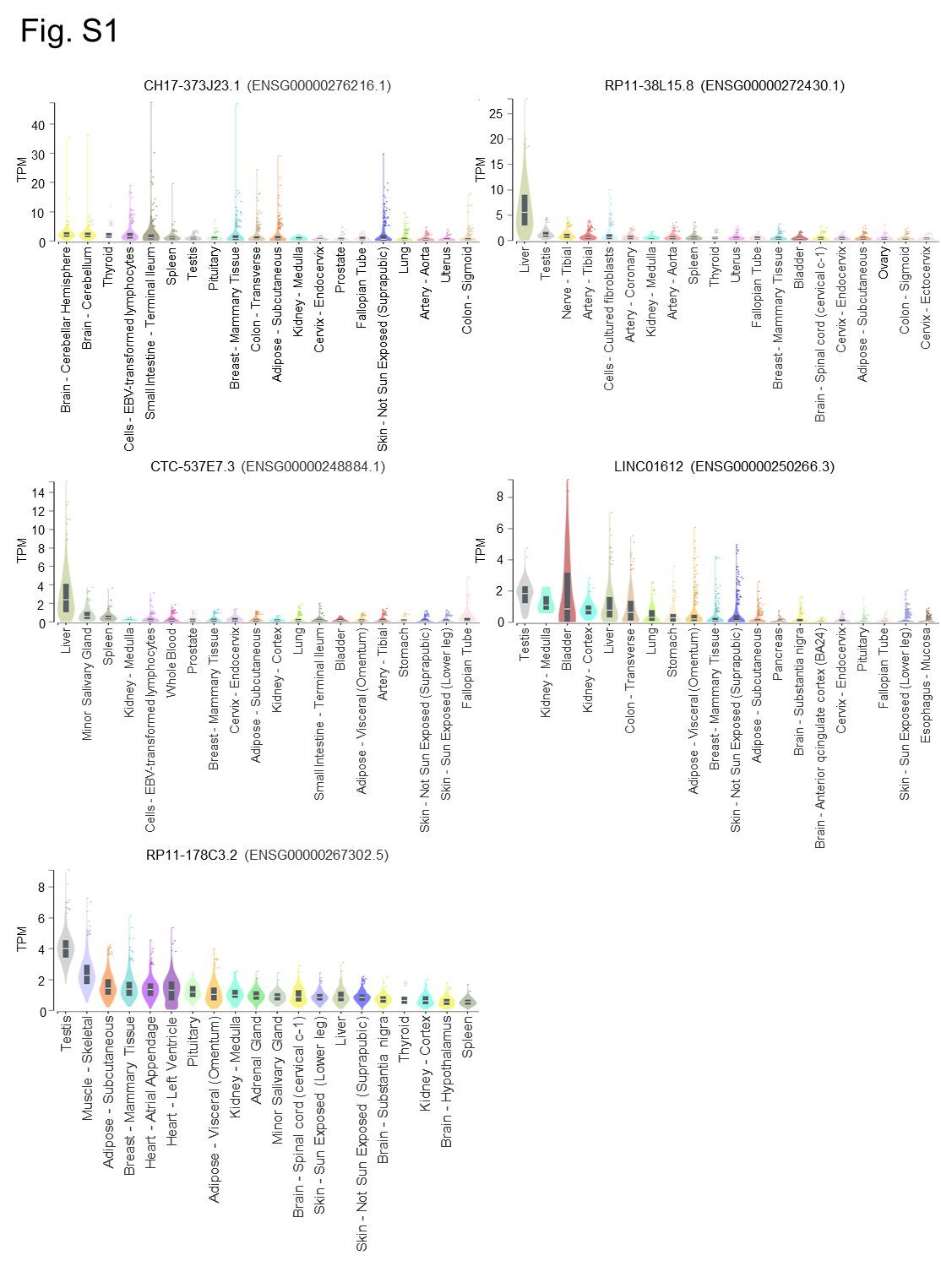


**Supplementary Fig. S1 Tissue-specific expression profiles of candidate lncRNAs.** Violin plots show the expression levels (TPM, Transcripts Per Million) of five candidate lncRNAs (*CH17-373J23.1*, *RP11-38L15.8*, *CTC-537E7.3*, *LINC01612*, and *RP11-178C3.2*) across various human tissues. Data were obtained from the GTEx portal and used to assess the liver specificity of candidates identified during the initial screening process.


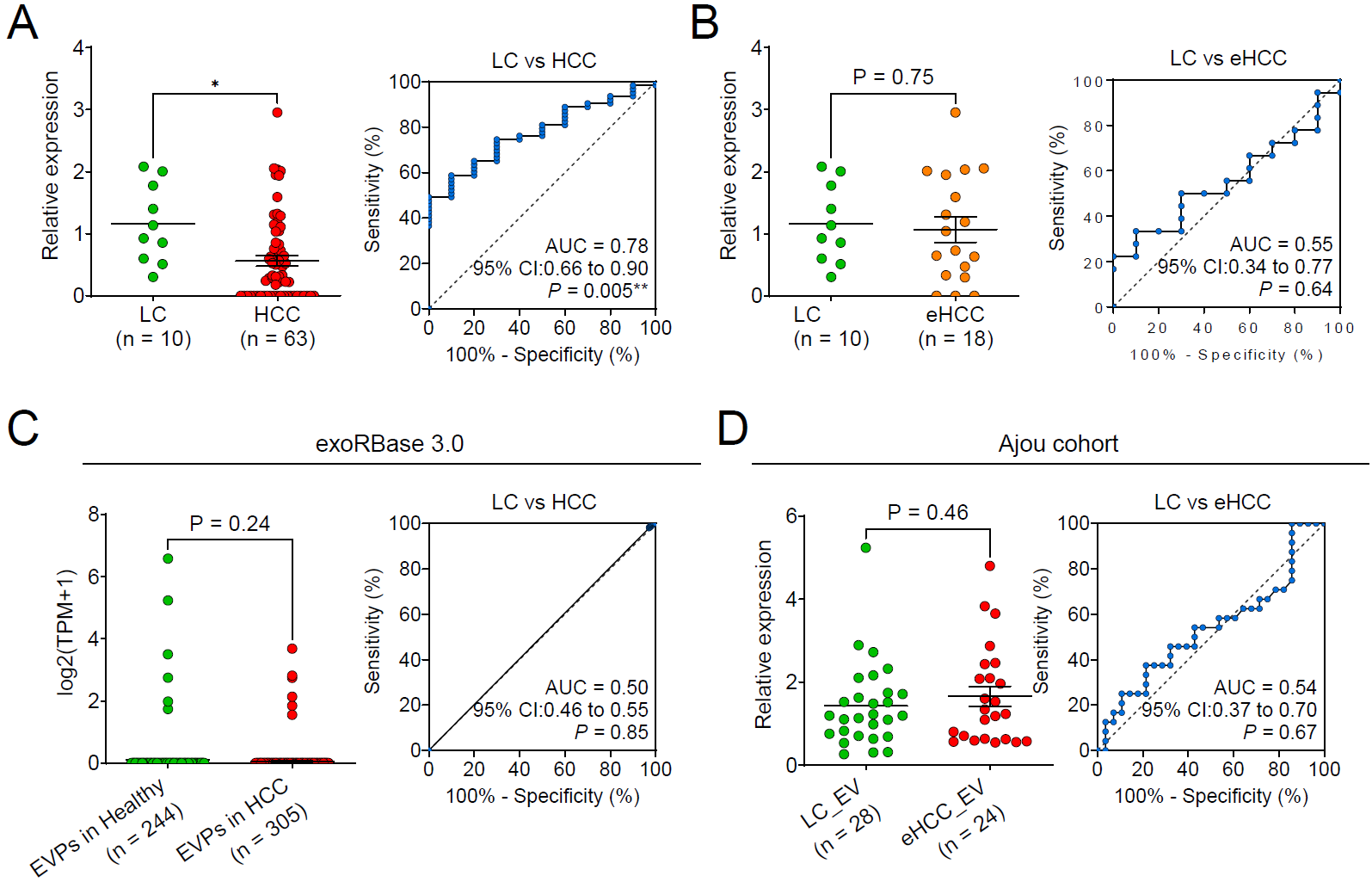


**Supplementary Fig. S2 Evaluation of *LUNAR* expression in cirrhosis versus HCC and in circulating extracellular vesicles.** (A) Comparative analysis of *LUNAR* expression in liver cirrhosis (LC, n = 10) and HCC (n = 63) from the GSE114564 dataset (left panel), with corresponding ROC curve analysis (right panel; AUC = 0.78, 95% CI: 0.66–0.90, *P* = 0.005). (B) Comparative analysis of *LUNAR* expression in LC (n = 10) and early HCC (eHCC, n = 18) from the GSE114564 dataset (left panel), with corresponding ROC curve analysis (right panel; AUC = 0.55, 95% CI: 0.34–0.77, *P* = 0.64), indicating limited discriminatory power between cirrhosis and early-stage HCC. (C) *LUNAR* expression levels in extracellular vesicle particles (EVPs) from healthy individuals (n = 244) and HCC patients (n = 305) obtained from the exoRBase 3.0 database (left panel), with corresponding ROC curve analysis (right panel; AUC = 0.50, 95% CI: 0.46–0.55, *P* = 0.85). (D) *LUNAR* expression in circulating EVs from LC (LC_EV, n = 28) and eHCC (eHCC_EV, n = 24) patients from the Ajou University Hospital cohort (left panel), with corresponding ROC curve analysis (right panel; AUC = 0.54, 95% CI: 0.37–0.70, *P* = 0.67). Data are presented as mean ± SD. Statistical comparisons were performed using unpaired Welch's t-test. Statistical significance levels (**P* < 0.05) are indicated where applicable.


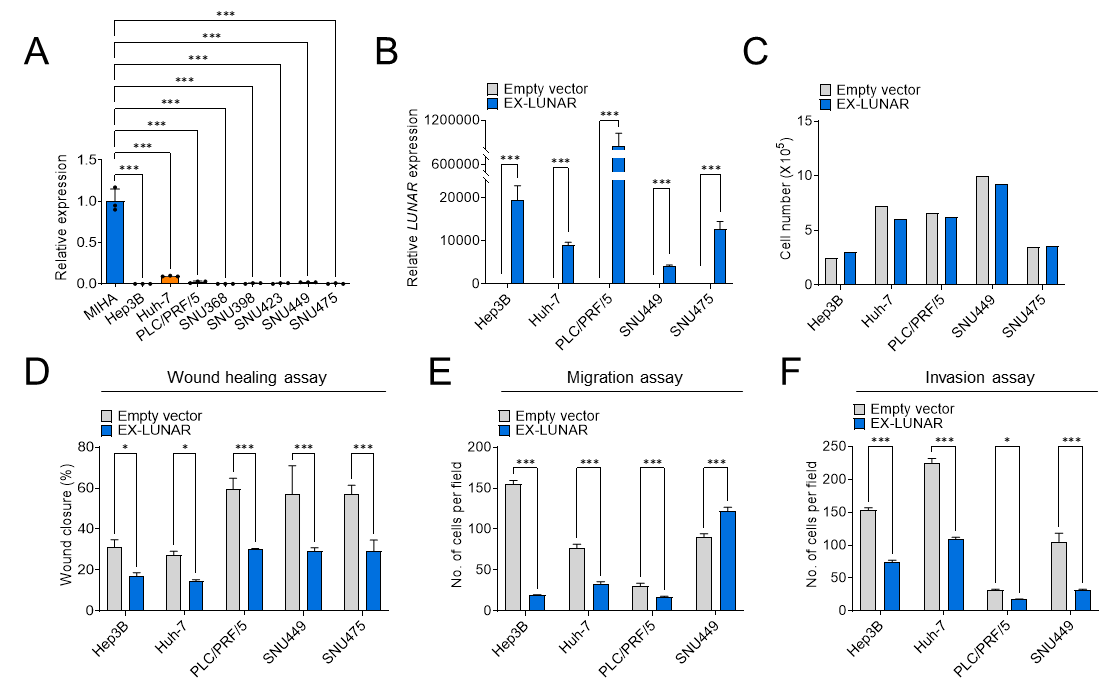


**Supplementary Fig. S3 Quantitative analysis of *in vitro* functional assays.** (A) qRT-PCR analysis of the basal expression levels of *LUNAR* in a normal hepatocyte cell line (MIHA) and eight HCC cell lines (Hep3B, Huh-7, PLC/PRF/5, SNU368, SNU398, SNU423, SNU449, and SNU475; n = 3 independent experiments). (B) qRT-PCR confirmation of robust *LUNAR* overexpression (EX-*LUNAR*) in five representative HCC cell lines (Hep3B, Huh-7, PLC/PRF/5, SNU449, and SNU475) following transfection, compared to the empty vector control (n = 3 independent experiments). (C) Quantification of cell numbers showing that *LUNAR* overexpression had no significant impact on cell proliferation in five tested HCC cell lines over 72 hours. (D) Quantitative analysis of the wound healing assay showing wound closure (%) upon *LUNAR* overexpression in five HCC cell lines (Hep3B, Huh-7, PLC/PRF/5, SNU449, and SNU475; n = 3 independent experiments). (E) Quantification of the transwell migration assay showing the number of migrated cells per field in four HCC cell lines (Hep3B, Huh-7, PLC/PRF/5, and SNU449; n = 3 independent experiments). (F) Quantification of the transwell invasion assay showing the number of invaded cells per field in four HCC cell lines (Hep3B, Huh-7, PLC/PRF/5, and SNU449; n = 3 independent experiments). Data are presented as mean ± SD. Statistical comparisons were performed using unpaired Welch's t-test. Statistical significance levels (**P* < 0.05, ****P* < 0.001) are indicated where applicable.


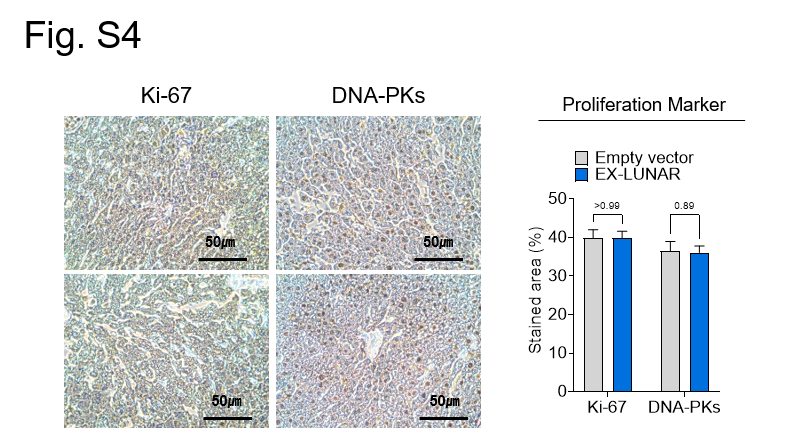


**Supplementary Fig. S4 Effect of *LUNAR* overexpression on tumor cell proliferation *in vivo*.** Representative IHC images showing the expression of proliferation markers Ki-67 and DNA-PKs in orthotopic tumor sections from mice injected with empty vector or EX-*LUNAR*-transfected Huh-7 cells (n = 3 per group). Scale bar = 50 μm. The bar chart on the right presents quantification of IHC staining intensity, showing no statistically significant difference between the two groups (Ki-67: *P* > 0.99; DNA-PKs: *P* = 0.89). Data are presented as mean ± SD. Statistical comparisons were performed using unpaired Welch's t-test.


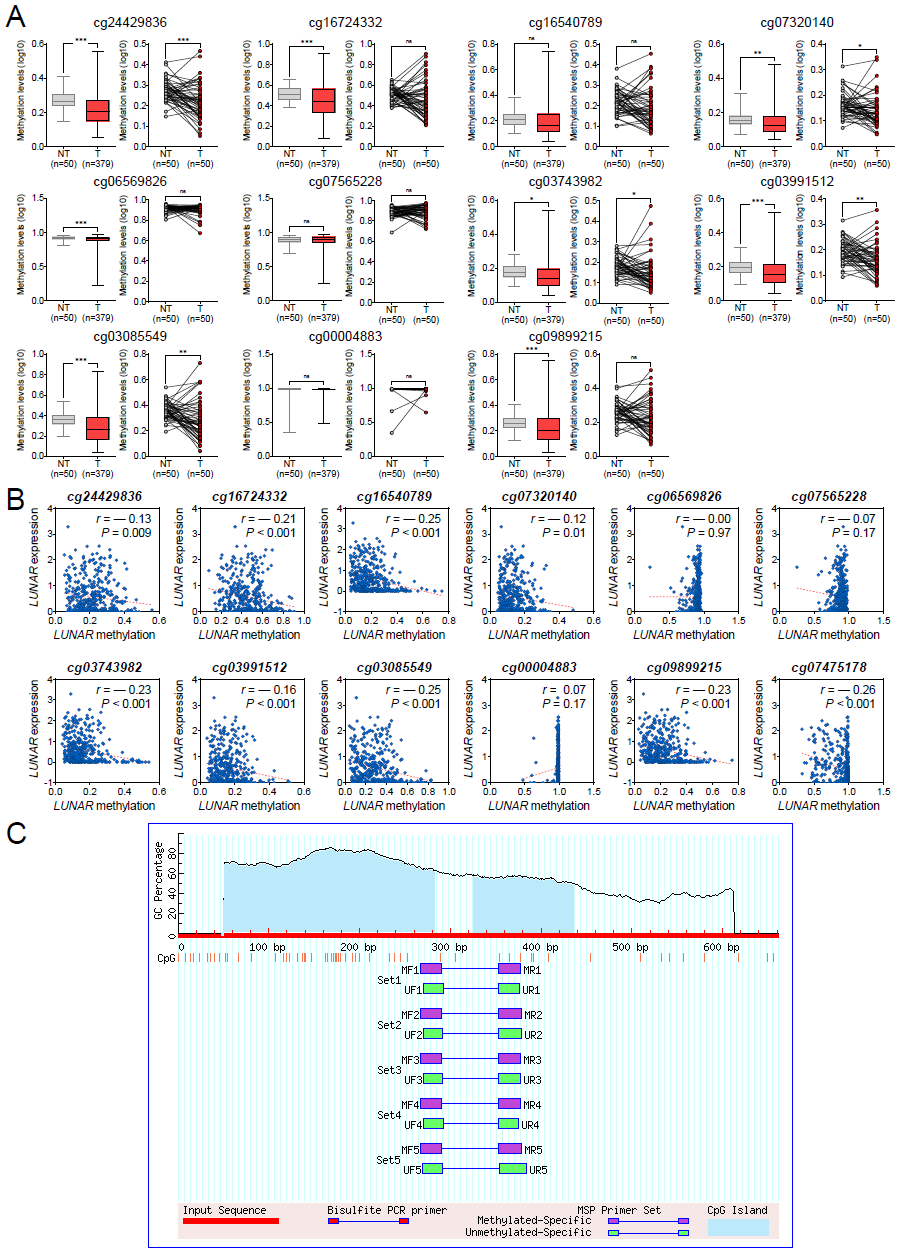


**Supplementary Fig. S5 Supporting data for the epigenetic regulation of *LUNAR*.** (A) Analysis of methylation levels for eleven CpG probes located at the *LUNAR* genomic locus, comparing non-tumor (NT, n = 50) and tumor (T, n = 379) tissues from the TCGA_LIHC dataset (box plots, left) and paired NT and T samples (n = 50 pairs, right). The methylation levels between paired NT and T samples were compared using paired Student’s t-test, whereas independent NT and T groups were compared using unpaired Welch’s t-test. (B) Pearson correlation analyses between *LUNAR* expression and methylation levels of all twelve CpG probes within the *LUNAR* genomic region in the TCGA_LIHC cohort. Scatter plots are shown for each probe, with correlation coefficients (r) and corresponding *P* values indicated. Among these, cg07475178 exhibited the strongest inverse correlation with *LUNAR* expression (r = –0.26, *P* < 0.001). (C) A schematic map of the *LUNAR* genomic region generated by the MethPrimer program, illustrating CpG islands and the specific locations of primer sets designed for quantitative methylation-specific PCR (qMSP) analysis. Statistical significance levels (**P* < 0.05, ***P* < 0.01, ****P* < 0.001) are indicated where applicable.


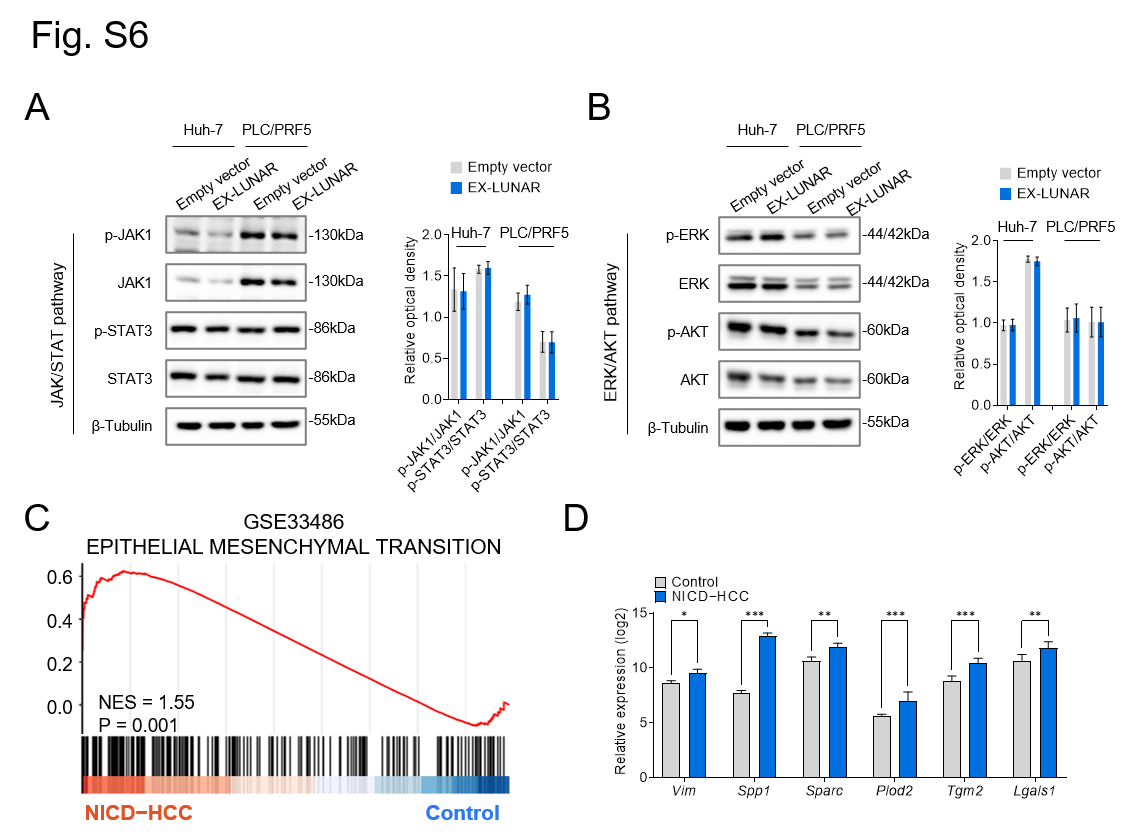


**Supplementary Fig. S6 Supporting analyses of JAK/STAT, ERK/AKT, and NOTCH-associated EMT signaling.** (A) Western blot analysis of JAK/STAT signaling pathway components (p-JAK1, JAK1, p-STAT3, and STAT3) in Huh-7 and PLC/PRF/5 cells transfected with an empty vector or EX-*LUNAR*; representative blots and quantified band densities normalized to β-Tubulin are shown (n = 3 independent experiments). No significant changes were observed in JAK/STAT pathway activity upon *LUNAR* overexpression. (B) Western blot analysis of ERK/AKT signaling pathway components (p-ERK, ERK, p-AKT, and AKT) in Huh-7 and PLC/PRF/5 cells transfected with an empty vector or EX-*LUNAR*; representative blots and quantified band densities normalized to β-Tubulin are shown (n = 3 independent experiments). No significant changes were observed in ERK/AKT pathway activity upon *LUNAR* overexpression. (C) Gene set enrichment analysis (GSEA) of the epithelial–mesenchymal transition (EMT) gene set in an independent NOTCH-driven mouse HCC dataset (GSE33486), comparing NICD-HCC tumors with control liver tissues. EMT-related gene sets were significantly enriched in NICD-driven tumors (normalized enrichment score [NES] = 1.55, *P* = 0.001). (D) qRT-PCR analysis of representative EMT-associated genes (*Vim*, *Spp1*, *Sparc*, *Plod2*, *Tgm2*, and *Lgals1*) in control and NICD-HCC liver tissues from the GSE33486 dataset. Data are presented as mean ± SD. Statistical comparisons were performed using unpaired Welch's t-test. Statistical significance levels (**P* < 0.05, ***P* < 0.01, ****P* < 0.001) are indicated where applicable.

Supplementary Tables

Supplementary Table S1. Antibodies used for Western blot and Immunohistochemistry.

| **Western blot** | | | | |
| --- | --- | --- | --- | --- |
| Antibody | Manufacturer | Dilution | Species | Cat No. |
| E-cadherin | BD Transduction Laboratories | 1:500 | Mouse | 610404 |
| ZO-1 | Thermo Fisher Scientific | 1:1000 | Mouse | 33-9100 |
| Fibronectin | Santa Cruz | 1:1000 | Mouse | sc-8422 |
| Vimentin | Cell signaling Technology | 1:1000 | Rabbit | 5741 |
| Nanog | Abcam | 1:1000 | Rabbit | ab109250 |
| CD133 | Abcam | 1:1000 | Rabbit | ab19898 |
| CD31 | Abcam | 1:1000 | Rabbit | ab134168 |
| VEGF | Santa Cruz | 1:1000 | Mouse | sc-7269 |
| JAK1 | Cell Signaling Technology | 1:1000 | Rabbit | 3344 |
| p-JAK1 | Cell Signaling Technology | 1:1000 | Rabbit | 74129 |
| STAT3 | Cell Signaling Technology | 1:1000 | Rabbit | 4904 |
| p-STAT3 | Cell Signaling Technology | 1:1000 | Rabbit | 9145 |
| ERK | Cell Signaling Technology | 1:1000 | Rabbit | 9102 |
| p-ERK | Cell Signaling Technology | 1:1000 | Rabbit | 9101 |
| AKT | Cell Signaling Technology | 1:1000 | Rabbit | 9272 |
| p-AKT | Cell Signaling Technology | 1:1000 | Rabbit | 9271 |
| Notch | Cell Signaling Technology | 1:1000 | Rabbit | 3608 |
| Cleaved Notch | Cell Signaling Technology | 1:1000 | Rabbit | 4147 |
| MAML1 | Cell Signaling Technology | 1:1000 | Rabbit | 12166 |
| RBPSUH | Cell Signaling Technology | 1:1000 | Rabbit | 5313 |
| β-Tubulin | Cell Signaling Technology | 1:1000 | Rabbit | 2146 |
| GAPDH | Santa cruz | 1:1000 | Mouse | sc-32233 |
| **Immunohistochemistry** | | | | |
| Antibody | Manufacture | Dilution | Species | Cat No |
| Ki-67 | Abcam | 1:50 | Rabbit | ab15580 |
| DNA-PKs | Cell signaling Technology | 1:100 | Rabbit | 38168 |
| E-cadherin | BD Transduction Laboratories | 1:100 | Mouse | 610404 |
| ZO-1 | Thermo Fisher Scientific | 1:200 | Mouse | 33-9100 |
| Fibronectin | Santa Cruz | 1:100 | Mouse | sc-8422 |
| Vimentin | Genetex | 1:500 | Rabbit | GTX100619 |
| Snail | Biorbyt | 1:250 | Rabbit | orb180479 |
| CD31 | Abcam | 1:500 | Rabbit | ab134168 |
| VEGF | Santa Cruz | 1:100 | Mouse | sc-7269 |
| HIF-1α | Cell signaling Technology | 1:200 | Rabbit | 48085 |
| CD133 | Abcam | 1:200 | Rabbit | ab19898 |

Supplementary Table S2. Differentially expressed lncRNAs identified across liver disease progression stages in GSE114564.

| Gene | Gene locus | Ensembl ID | NT mean | T mean | *P*-value | log_2_ FC |
| --- | --- | --- | --- | --- | --- | --- |
| CDC37L1-AS1 | chr9:4676599-4679502 | ENSG00000273061.1 | 4.66 | 1.85 | 6.35E-11 | -2.81 |
| RP11-393N21.2 | chr1:67988029-67998295 | ENSG00000235200.2 | 4.53 | 1.94 | 3.62E-12 | -2.59 |
| CH17-373J23.1 | chr1:148241464-148241811 | ENSG00000276216.1 | 6.83 | 4.62 | 1.04E-08 | -2.21 |
| RP11-327J17.2 | chr15:96533810-96883492 | ENSG00000259359.1 | 1.99 | 0.43 | 2.01E-08 | -1.56 |
| RP11-38L15.8 | chr10:46914144-46971400 | ENSG00000272430.1 | 1.91 | 0.44 | 1.03E-05 | -1.47 |
| RP11-1002K11.1 | chr8:32623642-32625477 | ENSG00000272327.1 | 2.01 | 0.55 | 0.000506 | -1.46 |
| RP11-444D3.1 | chr12:24366189-24715524 | ENSG00000255864.5 | 4.5 | 3.04 | 1.22E-09 | -1.45 |
| RP11-116D2.1 | chr2:21222184-21223789 | ENSG00000261012.2 | 5.51 | 4.06 | 9.09E-10 | -1.45 |
| CTC-537E7.3 | chr5:67726253-67730308 | ENSG00000248884.1 | 1.58 | 0.3 | 0.000107 | -1.28 |
| SNHG8 | chr4:119199863-119200978 | ENSG00000269893.6 | 6.97 | 5.7 | 1.54E-08 | -1.27 |
| U91319.1 | chr16:13340172-13656775 | ENSG00000262801.5 | 2.18 | 0.91 | 4.87E-06 | -1.27 |
| LINC01612 | chr4:171195069-171204230 | ENSG00000250266.1 | 1.62 | 0.36 | 0.000114 | -1.25 |
| DIO3OS | chr14:102018557-102026768 | ENSG00000258498.6 | 1.67 | 0.42 | 2.56E-06 | -1.24 |
| RP11-418I22.3 | chr16:9635901-9708751 | ENSG00000283003.1 | 2.13 | 0.91 | 2.01E-09 | -1.22 |
| RP11-622A1.2 | chr4:74374519-74399845 | ENSG00000250436.1 | 6.23 | 5.05 | 0.000589 | -1.17 |
| LINC00261 | chr20:22528308-22559280 | ENSG00000259974.2 | 5.89 | 4.73 | 6.03E-05 | -1.16 |
| RP11-168L7.1 | chr14:101798852-101800668 | ENSG00000258460.1 | 1.92 | 0.77 | 1.46E-05 | -1.15 |
| AC016768.1 | chr2:23240996-23421927 | ENSG00000232451.1 | 2.3 | 1.16 | 0.000155 | -1.13 |
| RMRP | chr9:35657747-35658015 | ENSG00000269900.3 | 17.14 | 16.05 | 4.25E-08 | -1.09 |
| RP11-252E2.2 | chr16:75153455-75178098 | ENSG00000261058.1 | 1.48 | 0.44 | 2.27E-07 | -1.04 |
| RP11-178C3.2 | chr17:57970406-58096336 | ENSG00000267302.5 | 1.9 | 0.9 | 8.17E-08 | -1 |
| RP11-81H3.2 | chr12:74526945-74796315 | ENSG00000251138.6 | 0.03 | 1.06 | 2.92E-06 | 1.03 |
| FLVCR1-AS1 | chr1:213025449-213031430 | ENSG00000198468.7 | 0.63 | 1.66 | 1.84E-08 | 1.03 |
| RP11-25H12.1 | chr4:66864563-67015730 | ENSG00000249413.2 | 0.01 | 1.04 | 4.89E-13 | 1.03 |
| RP11-844P9.2 | chr5:175564159-175626298 | ENSG00000248596.6 | 0.54 | 1.58 | 1.90E-08 | 1.04 |
| RP11-328J2.1 | chr7:119344609-119547429 | ENSG00000225546.5 | 0 | 1.13 | 8.16E-08 | 1.13 |
| CTC-261N6.2 | chr5:85676477-85677244 | ENSG00000250124.1 | 0.04 | 1.2 | 9.14E-09 | 1.16 |
| LINC01287 | chr7:153097004-153111048 | ENSG00000234722.3 | 0.05 | 1.23 | 0.000125 | 1.17 |
| LINC00511 | chr17:70319263-70636611 | ENSG00000227036.6 | 0.43 | 1.62 | 2.42E-06 | 1.18 |
| RP11-138J23.1 | chr5:103415611-103441104 | ENSG00000251026.1 | 0.03 | 1.27 | 8.57E-10 | 1.24 |
| RP11-19C24.1 | chr4:66864563-67015730 | ENSG00000272304.1 | 0.02 | 1.35 | 6.49E-14 | 1.33 |

***** NT, non-tumor tissue; T, tumor tissue; FC, fold change.
